# Four subgroups based on tau levels in Alzheimer’s disease observed in two independent cohorts

**DOI:** 10.1186/s13195-020-00713-3

**Published:** 2021-01-04

**Authors:** Flora H. Duits, Kirsten E. J. Wesenhagen, Laura Ekblad, Emma Wolters, Eline A. J. Willemse, ADNI, Philip Scheltens, Wiesje M. van der Flier, Charlotte E. Teunissen, Pieter Jelle Visser, Betty M. Tijms

**Affiliations:** 1 Department of Neurology, Alzheimer Center Amsterdam, Amsterdam Neuroscience, Vrije Universiteit Amsterdam, Amsterdam UMC, Amsterdam, the Netherlands; 2 Turku PET Centre, University of Turku and Turku University Hospital, Turku, Finland; 3 Department of Radiology & Nuclear Medicine, Amsterdam Neuroscience, Amsterdam UMC, Amsterdam Neuroscience, Amsterdam, Netherlands; 4 Department of Clinical Chemistry, Neurochemistry Laboratory, Amsterdam UMC, Amsterdam Neuroscience, Amsterdam, Netherlands; 5 Department of Epidemiology and Biostatistics, Amsterdam UMC, Amsterdam, The Netherlands; 6 Alzheimer Center Limburg, Department of Psychiatry & Neuropsychology, School of Mental Health and Neuroscience, Maastricht University, Maastricht, The Netherlands; 7 Division of Neurogeriatrics, Department of Neurobiology, Care Sciences and Society, Karolinska Institutet, Stockholm, Sweden

**Keywords:** Alzheimer’s disease, CSF tau, Gaussian mixture modelling, Prognosis

## Abstract

**Background:**

As Alzheimer’s disease (AD) pathology presents decades before dementia manifests, unbiased biomarker cut-points may more closely reflect presence of pathology than clinically defined cut-points. Currently, unbiased cerebrospinal fluid (CSF) tau cut-points are lacking.

**Methods:**

We investigated CSF t-tau and p-tau cut-points across the clinical spectrum using Gaussian mixture modelling, in two independent cohorts (Amsterdam Dementia Cohort and ADNI)*.*

**Results:**

Individuals with normal cognition (NC) (total *n* = 1111), mild cognitive impairment (MCI) (total *n* = 1213) and Alzheimer’s disease dementia (AD) (total *n* = 1524) were included. In both cohorts, four CSF t- and p-tau distributions and three corresponding cut-points were identified. Increasingly high tau subgroups were characterized by steeper MMSE decline and higher progression risk to AD (cohort/platform-dependent HR, t-tau 1.9–21.3; p-tau 2.2–9.5).

**Limitations:**

The number of subjects in some subgroups and subanalyses was small, especially in the highest tau subgroup and in tau PET analyses.

**Conclusions:**

In two independent cohorts, t-tau and p-tau levels showed four subgroups. Increasingly high tau subgroups were associated with faster clinical decline, suggesting our approach may aid in more precise prognoses.

## Background

Abnormal levels of amyloid-β 1-42 (Aβ42), total tau (t-tau) and tau phosphorylated at threonine 181 (p-tau-181) are biomarkers for the presence of Alzheimer’s disease (AD) pathology in the brain [1], and part of established research criteria for AD across the cognitive continuum [2, 3]. Classification schemes based on biomarkers depend on cut-points, and different approaches exist to determine such cut-points. The most often used traditional approach determines cut-points by optimizing the sensitivity and specificity to detect clinical AD-type dementia compared to controls [4–6]. However, approaches that use clinical labels as outcomes may not be optimal, because clinical labels do not optimally reflect the absence or presence of AD pathology: For example, almost 30% of cognitively intact individuals in their seventies have AD pathology [7], and up to 20% of clinical AD dementia cases do not show AD pathology at neuropathological examination [8–11]. As such, cut-point based on clinical labels can be biased.

Gaussian mixture modelling provides an approach to determine cut-points independent of clinical information [12]. This approach is based on the notion that the distribution of biomarker values in a population is a mixture of values belonging to subpopulations, i.e. normal and affected individuals. Previous studies using this approach have found a bimodal distribution of Aβ42 levels, of which the cut-point (i.e. the intersection of these distributions) was higher than clinically based cut-points, resulting in more sensitive detection of predementia AD [13–16]. As of yet, however, it remains unclear whether it is also possible to detect unbiased cut-points in t-tau and p-tau levels.

High t-tau levels in the cerebrospinal fluid (CSF) are thought to reflect neuronal degeneration or injury, and elevated t-tau levels can be found in the CSF in various conditions involving neuronal death, for example after an acute stroke. In contrast, p-tau-181 is presumed to reflect the formation of phosphorylated tau in the brain and to represent more specifically the formation of neurofibrillary tangles, one of the neuropathological hallmarks of AD [17, 18]. As tau pathology is a hallmark of AD, it can be hypothesized that similarly to amyloid, t- and p-tau levels may be a mixture of values belonging to normal and affected individuals, from which unbiased cut-points might be determined.

The objective of this study was to investigate whether subgroups can be identified in CSF t- and p-tau levels using Gaussian mixture modelling and to determine cut-points. We characterized tau subgroups in terms of clinical and biological characteristics and longitudinal trajectories of cognitive decline. We repeated analyses in the independent ADNI cohort to determine the robustness of the identified subgroups and tested stability of group membership by studying longitudinal changes in t-tau and p-tau levels. Finally, we compared subgroups on tau PET uptake that was available for a subset of individuals in ADNI.

## Methods

We investigated the existence of CSF t- and p-tau subgroups in data from two independent clinical cohorts. The memory clinic-based Amsterdam Dementia Cohort (ADC) was used for testing our hypothesis [19], and the Alzheimer’s Disease Neuroimaging Initiative (ADNI; www.adni-info.org) was used for validation of the results. ADNI started in 2003 as a public-private collaboration under the supervision of Principal Investigator Michael W. Weiner, MD. The primary goal of ADNI is to study whether serial magnetic resonance imaging (MRI), positron emission tomography (PET), other biological markers and clinical and neuropsychological measures can be combined to measure the progression of mild cognitive impairment (MCI) and early Alzheimer’s disease (AD). Please see www.adni-info.org for the latest information. The institutional review boards of all participating institutions approved the procedures for this study. Written informed consent was obtained from all participants or surrogates. Participant selection and CSF analyses are summarized below; for more detailed methods and information on Apolipoprotein E (APOE) genotyping and PET imaging, see Additional file 1.

### Participants

In short, patients from the ADC who visited our memory clinic between November 2000 and December 2016 were selected (*n* = 2724) if they had baseline CSF tau measurements available and had subjective cognitive decline (considered as normal cognition (NC)), mild cognitive impairment (MCI) or AD dementia. Participants from ADNI who had baseline CSF biomarkers available were selected (*n* = 1221) for the replication analyses if they met the study-specific criteria of NC, MCI or dementia. A subset of 619 individuals in ADNI (51%; 183 NC, 345 MCI and 91 with AD dementia) with available follow-up CSF measures were selected for longitudinal analyses.

### CSF biomarkers

In ADC, CSF biomarkers (β-amyloid_(1-42)_, hTAU-Ag, and phospo-tau 181P) were assessed with INNOTEST (Fujirebio, Ghent, Belgium) on a routine basis as described before [20]. In ADNI, CSF biomarkers were analysed using a multiplex xMAP Luminex platform (Luminex Corp) with immunoassay kit-based reagents (INNO-BIA Alzbio3; Innogenetics) [21] (*n* = 1213 participants), and on Elecsys (Roche, Basel, Switserland) [21] (*n* = 1193 participants, overlap with Luminex 98%).

### Statistical analysis

Gaussian mixture modelling was used to identify cut-points in the distribution of t-tau and p-tau values. First, the number of distributions that best described the data was determined with the R boot.comp function. This function sequentially tests increasing number of components in the data using parametric bootstrapping of the likelihood ratio (i.e. likelihood of x components vs. likelihood of having one more component, i.e. *x* + 1), until the null hypothesis cannot be rejected anymore (*p* > 0.05, i.e. no improvement of additional component for model fit). Then, we identified data-driven cut-points as the points where the lines of two fitted Gaussian distributions intersected. Using these cut-points, we labelled subjects according to tau subgroups. Next, within each cohort, we compared subgroups based on demographical, clinical and biological characteristics with ANOVA or chi-square tests, when appropriate. For a subset of individuals with available repeated mini-mental state examination (MMSE) and/or clinical follow-up, we further assessed whether subgroups showed differences in cognitive decline, stratifying subjects based on their baseline cognitive state (defined as NC, MCI or dementia). First, decline in MMSE (outcome) was assessed with linear mixed models using the R package “lmer4”, including the main terms time and tau subgroup, and interaction terms time*tau subgroup. For individuals without dementia at baseline, Cox proportional hazards models were used to compare the rate of progression from NC to MCI or AD dementia and from MCI to AD dementia between tau subgroups. We ran 5 models: (1) without covariates; (2) including age, sex and educational level; (3) model 2 + amyloid status; (4) model 3 + baseline cognitive state; and (5) model 4 + APOE-e4 carriership (dichotomous). For the Cox proportional hazards models, data from 945 subjects was available (357 normal and 588 MCI subjects). Subsets of individuals in ADNI also had repeated CSF tau measures available, for which we tested changes over time in tau subgroups, or had TAU PET available, for which we compared tau uptake according to Braak stages between subgroups. All analyses except for Cox proportional hazard analyses were stratified for baseline cognitive state, and adjusted for age and sex, and cognitive outcomes additionally for level of education [22]. In Cox proportional hazard analyses, no stratification for baseline cognitive state was performed due to small size of the resulting groups; instead, baseline cognitive state was added as additional covariate. All statistical analyses were performed in R version 3.6.1 “Action of the Toes”, mixture modelling was performed with the mixtools package (version 1.1.0), estimated marginal means and trends were computed with the R package “emmeans” v1.4, and sensitivity and specificity analyses with epiR v.1.0-15.

## Results

### Patient characteristics

Table 1 shows baseline characteristics of the ADC and ADNI cohorts. Compared to the ADC, subjects in the ADNI cohort were approximately 10 years older and had a lower prevalence of AD dementia and a higher prevalence of MCI. In ADC, subjects with NC were about 7 years younger compared to MCI and AD patients, and the NC and the MCI subjects were more often male than AD dementia subjects. In ADNI, MCI subjects were youngest, and MCI and AD dementia subjects were more often male than NC. In both cohorts, AD dementia subjects had lowest baseline MMSE scores, highest proportion of APOE e4 carriers, lowest levels of Aβ42, and highest levels of tau. MCI subjects had values in between NC subjects and AD dementia patients.

**Table 1:** Participant characteristics of the Amsterdam Dementia Cohort (ADC) and ADNI cohorts

	ADC			ADNI		
Characteristic	NC *N* = 740	MCI *N* = 591	AD dementia *N* = 1296	NC *N* = 371	MCI *N* = 622	AD dementia *N* = 228
MMSE, mean ± SD	28.2 ± 1.8	26.5 ± 2.4 ^a^	20.5 ± 5 ^c^	29.1 ± 1.2	27.7 ± 1.8 ^a^	23.3 ± 2 ^c^
Age, mean ± SD	59.6 ± 8.9	66.4 ± 8.2 ^a^	66.2 ± 8.1 ^a^	73.8 ± 5.9	72.4 ± 7.5 ^a^	74.9 ± 8.1 ^b^
Female, *n* (%)	306 (41.4%)	217 (36.7%) ^a^	674 (52%) ^c^	195 (52.6%)	255 (41%) ^a^	95 (41.7%) ^c^
APOE e4 carrier, *n* (%) NC	258 (36.3%)	287 (52.4%) ^a^	791 (65.3%) ^c^	103 (27.8%)	307 (49.4%) ^a^	154 (67.5%) ^c^
Innotest: T-tau (pg/ml), mean ± SD	296.4 ± 200.8	466.4 ± 303.6 ^a^	716.6 ± 417.1 ^c^	n.a.	n.a.	n.a.
Innotest: P-tau (pg/ml), mean ± SD	48.4 ± 22.7	66.8 ± 33.6 ^a^	87.6 ± 39.5 ^c^	n.a.	n.a.	n.a.
Innotest: Aβ42 (pg/ml), mean ± SD	1071.2 ± 246.9	859.1 ± 288.1 ^a^	648.4 ± 166.6 ^c^	n.a.	n.a.	n.a.
Innotest: Abnormal Aβ42 (< 813 pg/ml), *n* (%)	124 (16.8%)	326 (55.2%) ^a^	1173 (90.5%) ^c^	n.a.	n.a.	n.a.
Luminex T-tau (pg/ml), mean ± SD	n.a.	n.a.	n.a.	67.4 ± 32.8	90.4 ± 54.8 ^a^	126.6 ± 61.4 ^c^
Luminex P-tau (pg/ml), mean ± SD	n.a.	n.a.	n.a.	32.4 ± 18.8	39.2 ± 23.7 ^a^	51.6 ± 30.7 ^c^
Luminex Aβ42 (pg/ml), mean ± SD	n.a.	n.a.	n.a.	201.7 ± 51.9	171.2 ± 52.5 ^a^	139.6 ± 38.8 ^c^
Luminex Abnormal Aβ42 (< 192 pg/ml), n(%)	n.a.	n.a.	n.a.	156 (42%)	403 (64.8%) ^a^	210 (92.1%) ^c^
Elecsys T-tau (pg/ml), mean ± SD	n.a.	n.a.	n.a.	238.5 ± 90	284.9 ± 126.7 ^a^	370.2 ± 144.4 ^c^
Elecsys P-tau (pg/ml), mean ± SD	n.a.	n.a.	n.a.	21.9 ± 9.2	27.6 ± 14.4 ^a^	36.9 ± 15.7 ^c^
Elecsys Aβ42 (pg/ml), mean ± SD	n.a.	n.a.	n.a.	1337 ± 647.9	1020.7 ± 554.8 ^a^	694.6 ± 420.7 ^c^
Elecsys Abnormal Aβ42 (< 880 pg/ml), *n* (%)	n.a.	n.a.	n.a.	104 (28%)	325 (53%) ^a^	189 (85%) ^c^

*n.a.* not available, *NC* cognitively normal, *MCI* mild cognitive impairment, *AD* Alzheimer’s disease

^a^Differs from NC with *p* < .05

^b^Differs from MCI with *p* < .001

^c^Differs from MCI and NC with *p* < .001

### Gaussian mixture modelling reveals four subgroups

**Fig. 1:**
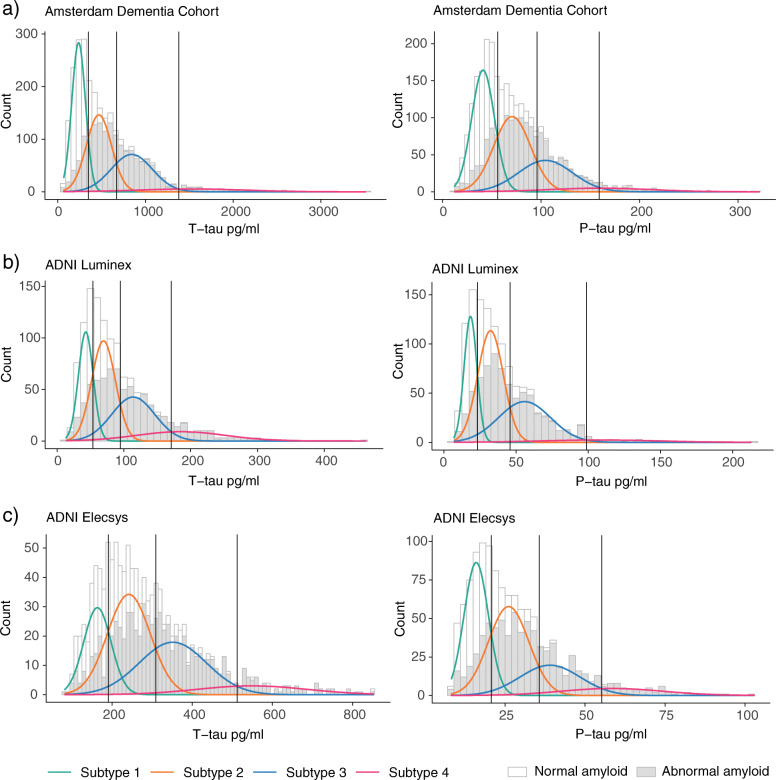
Tetramodal distributions in t-tau and p-tau levels in ADC and ADNI. Levels in ADC are shown in (**a**); levels in ADNI are shown in (**b**) for Luminex and (**c**) for Elecsys assay. Grey colours in the distributions reflect for those tau levels the number of individuals with abnormal amyloid levels

Mixture modeling showed that four distributions (i.e. a tetramodel distribution) best fitted the data for both t-tau and p-tau levels, with an optimal fit for four distributions (log-likelihood ratio for 3 vs. 4 distributions, for t-tau: 97.2, and p-tau: 28.3, both *p* < 0.001, no further improvement for 5 distributions: log-likelihood ratio for 5 vs 4 distributions, for t-tau: 3.9, and p-tau 15, both *p* > 0.05; see Additional file 2 for fit statistics of all fitted models, and Fig. 1a for a visualisation of the four distributions). In the ADC (using Innotest), this yielded three cutoffs (95% confidence interval (CI)), for t-tau—349 (304–382), 671 (582–834) and 1380 (1260–1505) pg/mL, and for p-tau—56 (46–60), 96 (71–121) and 159 (138–240) (for *n* per subgroup defined by cut-points, see Tables 2 and 3). The first cut-points for t-tau (349 pg/mL) and p-tau (56 pg/mL) were comparable to the t-tau and p-tau cut-points of 375 pg/ml and 52 pg/ml we previously reported [5], and showed similar sensitivity and specificity performance to distinguish between clinical AD dementia and controls (see Table 4 for sensitivity and specificity comparisons). Sensitivity and specificity for distinguishing NC vs MCI were also comparable to those resulting from the clinical cut-point (Table 4).

**Table 2:** Consistency of subgroup labelling between t-tau and p-tau (ADC and ADNI), and across platforms (ADNI)

Biomarker: Platform		ADC p-tau Innotest			
ADC T-tau: Innotest	Subgroup	1	2	3	4
	1	960	83	0	0
	2	140	661	58	0
	3	6	209	399	18
	4	0	0	33	60
		P-tau: Luminex			
T-tau: Luminex	Subgroup	1	2	3	4
	1	210	108	13	1
	2	116	261	89	2
	3	3	159	138	8
	4	0	10	76	19
		P-tau: Elecsys			
T-tau: Elecsys	Subgroup	1	2	3	4
	1	290	0	0	0
	2	186	309	0	0
	3	0	145	185	2
	4	0	0	14	61
		T-tau: Elecsys			
T-tau: Luminex	Subgroup	1	2	3	4
	1	256	70	1	0
	2	35	381	43	1
	3	0	44	255	4
	4	0	0	33	70
		P-tau: Elecsys			
P-tau: Luminex	Subgroup	1	2	3	4
	1	287	36	0	0
	2	167	296	63	2
	3	21	119	121	48
	4	0	1	13	12

**Table 3:** T-tau and p-tau subgroup comparisons on baseline characteristics

Biomarker: platform	Cohort	Characteristic	Cognitive state	Subgroup 1	Subgroup 2	Subgroup 3	Subgroup 4	*p* value 1 vs 2	*p* value 1 vs 3	*p* value 1 vs 4	*p* value 2 vs 3	*p* value 2 vs 4	*p* value 3 vs 4
T-tau: Innotest	ADC	*N*	All	1043	859	632	93	n.t.	n.t.	n.t.	n.t.	n.t.	n.t.
		T-tau pg/ml cutoffs	All	≤ 349	350–671	672–1380	> 1380	n.t.	n.t.	n.t.	n.t.	n.t.	n.t.
		*N* (%)	NC	576 (78%)	135 (18%)	24 (3%)	5 (1%)	n.t.	n.t.	n.t.	n.t.	n.t.	n.t.
			MCI	262 (44%)	207 (35%)	118 (20%)	4 (1%)	n.t.	n.t.	n.t.	n.t.	n.t.	n.t.
			AD dementia	205 (16%)	517 (40%)	490 (38%)	84 (6%)	n.t.	n.t.	n.t.	n.t.	n.t.	n.t.
		Abnormal ab42, *n* (%)	NC	57 (9.9%)	47 (34.8%)	16 (66.7%)	4 (80%)	2.71E−12	6.49E−15	7.84E−05	4.00E−02	6.73E−01	9.90E−01
			MCI	74 (28.2%)	140 (67.6%)	108 (91.5%)	4 (100%)	2.45E−16	6.64E−29	6.01E−02	1.31E−05	9.90E−01	9.90E−01
			AD dementia	146 (71.2%)	472 (91.3%)	474 (96.7%)	81 (96.4%)	5.86E−11	1.08E−21	2.76E−05	2.96E−03	9.83E−01	9.90E−01
		MMSE, mean ± SD	NC	28.2 ± 1.8	28.2 ± 1.6	27.4 ± 2.1	27.8 ± 1.9	1.00E+00	1.34E−01	9.65E−01	1.61E−01	9.62E−01	9.62E−01
			MCI	26.7 ± 2.4	26.6 ± 2.2	25.9 ± 2.8	25.2 ± 2.6	9.85E−01	2.53E−02	6.41E−01	7.27E−02	6.84E−01	9.47E−01
			AD dementia	21.3 ± 4.6	20.8 ± 5	20.2 ± 5.1	18.8 ± 5.1	5.53E−01	3.94E−02	7.13E−04	2.82E−01	5.16E−03	8.41E−02
		Age, mean ± SD	NC	58.2 ± 8.5	64.2 ± 8	66.1 ± 9.7	65.8 ± 11.7	0.00E+00	4.83E−05	1.86E−01	7.42E−01	9.75E−01	1.00E+00
			MCI	64 ± 8.4	67.9 ± 7.6	69.1 ± 7.1	69.9 ± 12	1.24E−06	5.96E−08	4.54E−01	5.28E−01	9.58E−01	9.97E−01
			AD dementia	66.5 ± 8	66.5 ± 7.9	65.7 ± 8.3	65.7 ± 8.1	1.00E+00	6.10E−01	8.52E−01	3.68E−01	8.11E−01	1.00E+00
		Female, *n* (%)	NC	234 (40.6%)	57 (42.2%)	11 (45.8%)	4 (80%)	9.90E−01	9.90E−01	9.90E−01	9.90E−01	9.90E−01	9.90E−01
			MCI	65 (24.8%)	83 (40.1%)	67 (56.8%)	2 (50%)	3.53E−03	1.71E−08	9.90E−01	3.21E−02	9.90E−01	9.90E−01
			AD dementia	81 (39.5%)	263 (50.9%)	277 (56.5%)	53 (63.1%)	4.51E−02	3.63E−04	2.58E−03	4.94E−01	2.98E−01	9.90E−01
		APOE e4 carrier, *n* (%)	NC	183 (33.1%)	58 (45%)	15 (62.5%)	2 (40%)	8.88E−02	3.56E−02	9.90E−01	1.00E+00	9.90E−01	9.90E−01
			MCI	92 (36.9%)	115 (59.6%)	77 (75.5%)	3 (75%)	2.15E−05	6.98E−10	9.90E−01	5.64E−02	1.00E+00	9.90E−01
			AD dementia	113 (58.9%)	313 (65.3%)	310 (67.5%)	55 (67.1%)	8.18E−01	2.55E−01	9.90E−01	1.00E+00	1.00E+00	9.90E−01
T-tau: Luminex	ADNI	*N*	All	335	468	310	108	n.t.	n.t.	n.t.	n.t.	n.t.	n.t.
		T-tau pg/ml cutoffs	All	≤ 54	55–95	96–171	> 171 s	n.t.	n.t.	n.t.	n.t.	n.t.	n.t.
		*N* (%)	NC	154 (42%)	151 (41%)	63 (17%)	3 (1%)	n.t.	n.t.	n.t.	n.t.	n.t.	n.t.
			MCI	168 (27%)	249 (40%)	146 (23%)	59 (9%)	n.t.	n.t.	n.t.	n.t.	n.t.	n.t.
			AD dementia	13 (6%)	68 (30%)	101 (44%)	46 (20%)	n.t.	n.t.	n.t.	n.t.	n.t.	n.t.
		Abnormal ab42, *n* (%)	NC	54 (35.1%)	55 (36.4%)	45 (71.4%)	2 (66.7%)	9.90E−01	1.34E−05	9.90E−01	3.58E−05	9.90E−01	9.90E−01
			MCI	53 (31.5%)	155 (62.2%)	138 (94.5%)	57 (96.6%)	8.68E−09	9.22E−29	1.73E−16	2.12E−11	4.08E−06	9.90E−01
			AD dementia	9 (69.2%)	59 (86.8%)	97 (96%)	45 (97.8%)	9.90E−01	1.70E−02	4.10E−02	3.26E−01	5.23E−01	9.90E−01
		MMSE, mean ± SD	NC	29.1 ± 1.2	29 ± 1	29.1 ± 1.4	29 ± 1	9.87E−01	1.00E+00	1.00E+00	9.97E−01	1.00E+00	1.00E+00
			MCI	28.3 ± 1.6	27.8 ± 1.8	27.3 ± 1.8	26.8 ± 1.9	2.27E−02	1.41E−05	6.60E−07	7.24E−02	1.66E−03	2.86E−01
			AD dementia	24.4 ± 1.3	23.6 ± 2	23 ± 2	23.3 ± 2	5.75E−01	1.02E−01	2.87E−01	2.60E−01	8.11E−01	9.11E−01
		Age, mean ± SD	NC	72.5 ± 5.6	74 ± 5.8	76.3 ± 6.2	74.1 ± 3.4	9.69E−02	5.53E−05	9.64E−01	3.49E−02	1.00E+00	9.10E−01
			MCI	70.7 ± 7.5	72.6 ± 7.6	73.7 ± 7.4	73.3 ± 7	5.69E−02	2.46E−03	1.15E−01	4.89E−01	9.34E−01	9.78E−01
			AD dementia	78.4 ± 6.6	75.6 ± 8.7	75.1 ± 7.5	72.2 ± 8.6	6.66E−01	4.98E−01	6.62E−02	9.72E−01	1.08E−01	1.73E−01
		Female, *n* (%)	NC	81 (52.6%)	78 (51.7%)	33 (52.4%)	3 (100%)	9.90E−01	9.90E−01	9.90E−01	9.90E−01	9.90E−01	9.90E−01
			MCI	68 (40.5%)	89 (35.7%)	64 (43.8%)	34 (57.6%)	1.00E+00	9.90E−01	2.01E−01	8.23E−01	1.98E−02	6.12E−01
			AD dementia	3 (23.1%)	18 (26.5%)	45 (44.6%)	29 (63%)	1.00E+00	9.90E−01	1.51E−01	1.58E−01	1.30E−03	3.44E−01
		APOE e4 carrier, *n* (%)	NC	32 (20.8%)	44 (29.1%)	26 (41.3%)	1 (33.3%)	7.19E−01	2.05E−02	9.90E−01	7.07E−01	1.00E+00	9.90E−01
			MCI	43 (25.6%)	117 (47%)	103 (70.5%)	44 (74.6%)	1.01E−04	2.45E−14	4.77E−10	5.27E−05	1.46E−03	9.90E−01
			AD dementia	7 (53.8%)	43 (63.2%)	75 (74.3%)	29 (63%)	9.90E−01	9.90E−01	1.00E+00	9.90E−01	9.90E−01	9.90E−01
T-tau: Elecsys	ADNI	*N*	All	291	495	332	75	n.t.	n.t.	n.t.	n.t.	n.t.	n.t.
		T-tau pg/ml cutoffs	All	≤ 192	193–311	312–514	> 514	n.t.	n.t.	n.t.	n.t.	n.t.	n.t.
		*N* (%)	NC	126 (35%)	164 (45%)	69 (19%)	4 (1%)	n.t.	n.t.	n.t.	n.t.	n.t.	n.t.
			MCI	152 (25%)	258 (42%)	157 (26%)	41 (7%)	n.t.	n.t.	n.t.	n.t.	n.t.	n.t.
			AD dementia	13 (6%)	73 (33%)	106 (48%)	30 (14%)	n.t.	n.t.	n.t.	n.t.	n.t.	n.t.
		Abnormal ab42, *n* (%)	NC	40 (31.7%)	67 (40.9%)	40 (58%)	3 (75%)	8.49E−01	3.93E−03	9.90E−01	1.47E−01	9.90E−01	9.90E−01
			MCI	52 (34.2%)	153 (59.3%)	146 (93%)	40 (97.6%)	9.25E−06	1.06E−25	1.23E−11	1.67E−12	2.77E−05	9.90E−01
			AD dementia	8 (61.5%)	67 (91.8%)	100 (94.3%)	29 (96.7%)	6.33E−02	4.91E−03	6.03E−02	9.90E−01	9.90E−01	9.90E−01
		MMSE, mean ± SD	NC	29.2 ± 1.1	29 ± 1.1	29 ± 1.4	29.2 ± 1	5.42E−01	8.40E−01	9.99E−01	9.95E−01	9.66E−01	9.79E−01
			MCI	28.1 ± 1.7	27.9 ± 1.8	27.2 ± 1.8	27.1 ± 1.8	6.43E−01	1.70E−05	6.98E−03	2.46E−04	3.86E−02	9.98E−01
			AD dementia	24.5 ± 1.5	23.5 ± 1.9	23 ± 2.1	23.5 ± 1.9	3.88E−01	7.21E−02	4.57E−01	3.92E−01	1.00E+00	6.83E−01
		Age, mean ± SD	NC	71.6 ± 5.5	74.4 ± 5.8	76.1 ± 6.2	76.7 ± 6	3.60E−04	1.86E−06	2.95E−01	1.56E−01	8.47E−01	9.96E−01
			MCI	70.9 ± 7.9	72.4 ± 7.4	73.9 ± 7.4	71.7 ± 6.9	2.02E−01	3.72E−03	9.26E−01	2.48E−01	9.47E−01	3.81E−01
			AD dementia	77.7 ± 7.3	75.6 ± 8.1	74.3 ± 8.1	73.5 ± 8.7	8.13E−01	4.81E−01	4.09E−01	7.40E−01	6.61E−01	9.69E−01
		Female, *n* (%)	NC	69 (54.8%)	80 (48.8%)	37 (53.6%)	4 (100%)	9.90E−01	9.90E−01	9.90E−01	9.90E−01	7.74E−01	9.90E−01
			MCI	55 (36.2%)	99 (38.4%)	72 (45.9%)	23 (56.1%)	9.90E−01	6.41E−01	2.01E−01	9.69E−01	2.90E−01	9.90E−01
			AD dementia	3 (23.1%)	23 (31.5%)	51 (48.1%)	17 (56.7%)	9.90E−01	9.40E−01	5.40E−01	2.35E−01	1.86E−01	9.90E−01
		APOE e4 carrier, *n* (%)	NC	26 (20.6%)	45 (27.4%)	29 (42%)	1 (25%)	9.90E−01	1.58E−02	1.00E+00	2.54E−01	9.90E−01	9.90E−01
			MCI	41 (27%)	120 (46.5%)	105 (66.9%)	32 (78%)	8.40E−04	2.90E−11	3.90E−08	4.95E−04	2.03E−03	9.90E−01
			AD dementia	5 (38.5%)	51 (69.9%)	75 (70.8%)	19 (63.3%)	3.67E−01	2.55E−01	9.90E−01	9.90E−01	9.90E−01	9.90E−01
P-tau: Innotest	ADC	*N*	All	1106	953	490	78	n.t.	n.t.	n.t.	n.t.	n.t.	n.t.
		P-tau pg/ml cutoffs	All	≤ 56	57–96	97–159	> 159	n.t.	n.t.	n.t.	n.t.	n.t.	n.t.
		*N* (%)	NC	564 (76%)	153 (21%)	19 (3%)	4 (1%)	n.t.	n.t.	n.t.	n.t.	n.t.	n.t.
			MCI	272 (46%)	214 (36%)	98 (17%)	7 (1%)	n.t.	n.t.	n.t.	n.t.	n.t.	n.t.
			AD dementia	270 (21%)	586 (45%)	373 (29%)	67 (5%)	n.t.	n.t.	n.t.	n.t.	n.t.	n.t.
		Abnormal ab42, *n* (%)	NC	61 (10.8%)	44 (28.8%)	16 (84.2%)	3 (75%)	3.22E−07	2.14E−18	6.88E−03	3.57E−05	8.98E−01	9.90E−01
			MCI	78 (28.7%)	153 (71.5%)	89 (90.8%)	6 (85.7%)	9.08E−20	6.37E−25	2.78E−02	1.57E−03	9.90E−01	9.90E−01
			AD dementia	204 (75.6%)	546 (93.2%)	358 (96%)	65 (97%)	4.83E−12	2.03E−13	1.07E−03	5.59E−01	9.90E−01	9.90E−01
		MMSE, mean ± SD	NC	28.2 ± 1.8	28.1 ± 1.6	27.9 ± 2.1	27.5 ± 1.7	9.99E−01	9.53E−01	8.79E−01	9.71E−01	8.94E−01	9.68E−01
			MCI	26.7 ± 2.3	26.6 ± 2.4	25.7 ± 2.7	27.1 ± 2.1	9.88E−01	1.99E−03	9.60E−01	7.13E−03	9.40E−01	3.93E−01
			AD dementia	20.9 ± 5	20.7 ± 4.9	20.3 ± 5.2	19 ± 4.8	9.11E−01	4.53E−01	2.98E−02	7.26E−01	5.43E−02	2.07E−01
		Age, mean ± SD	NC	58.3 ± 8.6	63.1 ± 8.3	67.1 ± 6.5	68.3 ± 16.8	7.58E−09	7.08E−05	9.30E−02	2.07E−01	6.19E−01	9.94E−01
			MCI	64.1 ± 8.2	68 ± 7.9	69.3 ± 6.8	70.2 ± 10.1	3.55E−07	1.67E−07	1.77E−01	5.48E−01	8.89E−01	9.91E−01
			AD dementia	66.7 ± 7.8	66.1 ± 8.1	65.6 ± 8	67.4 ± 9.2	7.76E−01	3.62E−01	9.13E−01	8.01E−01	6.02E−01	3.47E−01
		Female, *n* (%)	NC	228 (40.4%)	67 (43.8%)	9 (47.4%)	2 (50%)	9.90E−01	1.00E+00	9.90E−01	9.90E−01	9.90E−01	9.90E−01
			MCI	73 (26.8%)	85 (39.7%)	54 (55.1%)	5 (71.4%)	2.15E−02	4.97E−06	1.80E−01	9.45E−02	9.90E−01	9.90E−01
			AD dementia	118 (43.7%)	302 (51.5%)	215 (57.6%)	39 (58.2%)	2.38E−01	3.89E−03	2.77E−01	4.48E−01	9.90E−01	9.90E−01
		APOE e4 carrier, *n* (%)	NC	181 (33.3%)	62 (43.1%)	14 (73.7%)	1 (25%)	2.22E−01	4.13E−03	9.90E−01	1.39E−01	9.90E−01	9.90E−01
			MCI	90 (35%)	133 (66.2%)	60 (71.4%)	4 (66.7%)	4.10E−10	6.79E−08	9.90E−01	9.90E−01	9.90E−01	9.90E−01
			AD dementia	148 (59.2%)	367 (66.6%)	228 (65.7%)	48 (75%)	3.09E−01	7.44E−01	1.74E−01	9.90E−01	9.90E−01	9.90E−01
P-tau: Luminex	ADNI	*N*	All	329	538	316	30	n.t.	n.t.	n.t.	n.t.	n.t.	n.t.
		P-tau pg/ml cutoffs	All	≤ 23	24–46	47–99	> 99	n.t.	n.t.	n.t.	n.t.	n.t.	n.t.
		*N* (%)	NC	138 (37%)	166 (45%)	62 (17%)	3 (1%)	n.t.	n.t.	n.t.	n.t.	n.t.	n.t.
			MCI	173 (28%)	266 (43%)	166 (27%)	13 (2%)	n.t.	n.t.	n.t.	n.t.	n.t.	n.t.
			AD dementia	18 (8%)	106 (47%)	88 (39%)	14 (6%)	n.t.	n.t.	n.t.	n.t.	n.t.	n.t.
		Abnormal ab42, *n* (%)	NC	41 (29.7%)	66 (39.8%)	44 (71%)	3 (100%)	5.28E−01	6.79E−07	2.93E−01	3.11E−04	7.84E−01	9.90E−01
			MCI	47 (27.2%)	184 (69.2%)	155 (93.4%)	13 (100%)	9.96E−17	4.99E−34	1.93E−06	3.28E−08	2.30E−01	9.90E−01
			AD dementia	11 (61.1%)	98 (92.5%)	85 (96.6%)	14 (100%)	4.36E−03	1.28E−04	1.63E−01	9.90E−01	9.90E−01	9.90E−01
		MMSE, mean ± SD	NC	29.1 ± 1.2	29 ± 1.2	29.1 ± 1	30 ± 0	9.35E−01	1.00E+00	5.35E−01	9.83E−01	4.60E−01	5.32E−01
			MCI	28.2 ± 1.6	27.5 ± 1.9	27.5 ± 1.8	27 ± 1.5	2.41E−04	1.71E−03	7.75E−02	1.00E+00	7.42E−01	7.33E−01
			AD dementia	23.6 ± 1.9	23.3 ± 2	23.3 ± 2.1	23.6 ± 1.8	9.56E−01	9.15E−01	1.00E+00	9.94E−01	9.80E−01	9.55E−01
		Age, mean ± SD	NC	73 ± 5.3	73.7 ± 6.5	75.4 ± 5.6	72.8 ± 2.5	7.76E−01	4.23E−02	1.00E+00	1.98E−01	9.94E−01	8.74E−01
			MCI	71.7 ± 7.7	73 ± 7.8	72.6 ± 6.9	69.2 ± 7.8	2.58E−01	6.72E−01	6.78E−01	9.41E−01	2.92E−01	4.12E−01
			AD dementia	80.6 ± 7.8	76 ± 7.4	72.9 ± 8.5	72.4 ± 5.3	9.87E−02	9.25E−04	1.76E−02	2.83E−02	3.57E−01	9.96E−01
		Female, *n* (%)	NC	69 (50%)	88 (53%)	33 (53.2%)	3 (100%)	9.90E−01	9.90E−01	9.90E−01	9.90E−01	9.90E−01	9.90E−01
			MCI	65 (37.6%)	108 (40.6%)	73 (44%)	8 (61.5%)	9.90E−01	9.90E−01	9.47E−01	9.90E−01	9.90E−01	9.90E−01
			AD dementia	4 (22.2%)	43 (40.6%)	36 (40.9%)	10 (71.4%)	9.90E−01	9.90E−01	9.20E−02	9.90E−01	3.45E−01	3.92E−01
		APOE e4 carrier, *n* (%)	NC	28 (20.3%)	43 (25.9%)	29 (46.8%)	2 (66.7%)	9.90E−01	1.47E−03	9.90E−01	2.57E−02	9.90E−01	9.90E−01
			MCI	49 (28.3%)	132 (49.6%)	115 (69.3%)	10 (76.9%)	8.90E−05	6.32E−13	5.36E−03	5.41E−04	6.08E−01	9.90E−01
			AD dementia	7 (38.9%)	73 (68.9%)	61 (69.3%)	12 (85.7%)	1.71E−01	1.74E−01	1.24E−01	9.90E−01	9.90E−01	9.90E−01
P-tau: Elecsys	ADNI	*N*	All	467	454	199	63	n.t.	n.t.	n.t.	n.t.	n.t.	n.t.
		P-tau pg/ml cutoffs	All	≤ 21	22–36	37–55	> 55	n.t.	n.t.	n.t.	n.t.	n.t.	n.t.
		*N* (%)	NC	208 (57%)	127 (35%)	24 (7%)	3 (1%)	n.t.	n.t.	n.t.	n.t.	n.t.	n.t.
			MCI	241 (40%)	229 (38%)	105 (17%)	33 (5%)	n.t.	n.t.	n.t.	n.t.	n.t.	n.t.
			AD dementia	27 (12%)	98 (44%)	70 (32%)	27 (12%)	n.t.	n.t.	n.t.	n.t.	n.t.	n.t.
		Abnormal ab42, *n* (%)	NC	63 (30.3%)	61 (48%)	23 (95.8%)	2 (66.7%)	9.91E−03	7.59E−09	1.00E+00	2.49E−04	1.00E+00	1.00E+00
			MCI	83 (34.4%)	173 (75.5%)	102 (97.1%)	33 (100%)	5.17E−18	1.25E−25	2.03E−11	1.99E−05	1.75E−02	1.00E+00
			AD dementia	18 (66.7%)	92 (93.9%)	68 (97.1%)	26 (96.3%)	2.61E−03	6.13E−04	8.52E−02	1.00E+00	1.00E+00	1.00E+00
		MMSE, mean ± SD	NC	29.1 ± 1.2	28.9 ± 1.2	29.5 ± 0.8	29 ± 1	9.35E−01	1.00E+00	5.35E−01	9.83E−01	4.60E−01	5.32E−01
			MCI	28.1 ± 1.7	27.7 ± 1.8	27.2 ± 1.8	26.9 ± 1.9	2.41E−04	1.71E−03	7.75E−02	1.00E+00	7.42E−01	7.33E−01
			AD dementia	24 ± 1.7	23.3 ± 2	23 ± 2.1	23.6 ± 1.8	9.56E−01	9.15E−01	1.00E+00	9.94E−01	9.80E−01	9.55E−01
		Age, mean ± SD	NC	72.5 ± 5.5	75.2 ± 6.3	77.3 ± 5.5	74.1 ± 3.4	7.76E−01	4.23E−02	1.00E+00	1.98E−01	9.94E−01	8.74E−01
			MCI	70.9 ± 7.6	73.4 ± 7.6	73.4 ± 7.3	72.6 ± 7.1	2.58E−01	6.72E−01	6.78E−01	9.41E−01	2.92E−01	4.12E−01
			AD dementia	78.1 ± 7.7	75.2 ± 7.7	73.8 ± 8.4	72.9 ± 8.7	9.87E−02	9.25E−04	1.76E−02	2.83E−02	3.57E−01	9.96E−01
		Female, *n* (%)	NC	101 (48.6%)	75 (59.1%)	11 (45.8%)	3 (100%)	4.77E−01	1.00E+00	1.00E+00	1.00E+00	1.00E+00	1.00E+00
			MCI	94 (39%)	89 (38.9%)	46 (43.8%)	20 (60.6%)	1.00E+00	1.00E+00	1.79E−01	1.00E+00	1.76E−01	8.25E−01
			AD dementia	7 (25.9%)	35 (35.7%)	37 (52.9%)	15 (55.6%)	1.00E+00	1.84E−01	3.15E−01	2.39E−01	6.04E−01	1.00E+00
		APOE e4 carrier, *n* (%)	NC	45 (21.6%)	41 (32.3%)	14 (58.3%)	1 (33.3%)	2.51E−01	1.50E−03	1.00E−03	1.66E−01	1.00E+00	1.00E+00
			MCI	72 (29.9%)	122 (53.3%)	79 (75.2%)	25 (75.8%)	2.56E−06	7.89E−14	3.92E−06	1.36E−03	1.48E−01	1.00E+00
			AD dementia	10 (37%)	72 (73.5%)	52 (74.3%)	16 (59.3%)	5.81E−03	8.60E−03	1.00E+00	1.00E−03	1.00E+00	1.00E+00

All pairwise comparisons are Tukey HSD adjusted for multiple testing

*n.t.* not tested

**Table 4:** Sensitivity and specificity for clinical comparisons

			NC vs AD-type dementia				NC vs MCI			
			First cut-point		Cut-point (literature)		First cut-point		Cut-point (literature)	
Dataset	First cut-point	Cut-point (literature)	Sensitivity	Specificity	Sensitivity	Specificity	Sensitivity	Specificity	Sensitivity	Specificity
T-tau										
ADC: Innotest	349	375^a^	0.84 (0.8–0.86)	0.78 (0.74–0.82)	0.81 (0.78–0.84)	0.82 (0.78–0.86)	0.56 (0.52–0.6)	0.78 (0.75–0.81)	0.52 (0.47–0.56)	0.82 (0.79–0.84)
ADNI: Luminex	54	93^b^	0.95 (0.9–0.98)	0.4 (0.33–0.48)	0.64 (0.55–0.72)	0.81 (0.74–0.86)	0.73 (0.69–0.76)	0.42 (0.36–0.47)	0.35 (0.31–0.39)	0.81 (0.76–0.84)
ADNI: Elecsys	192	300^c^	0.94 (0.88–0.97)	0.33 (0.26–0.4)	0.64 (0.55–0.72)	0.78 (0.71–0.84)	0.76 (0.72–0.79)	0.34 (0.29–0.39)	0.37 (0.33–0.4)	0.77 (0.72–0.81)
P-tau										
ADC: Innotest	56	52^a^	0.78 (0.75–0.81)	0.77 (0.72–0.81)	0.83 (0.8–0.86)	0.72 (0.68–0.77)	0.54 (0.5–0.58)	0.76 (0.73–0.79)	0.59 (0.55–0.63)	0.7 (0.67–0.73)
ADNI: Luminex	23	23^b^	0.9 (0.84–0.95)	0.4 (0.33–0.47)	0.9 (0.84–0.95)	0.4 (0.33–0.48)	0.72 (0.68–0.76)	0.37 (0.32–0.42)	0.72 (0.68–0.76)	0.37 (0.32–0.43)
ADNI: Elecsys	21	24^c^	0.84 (0.76–0.9)	0.56 (0.48–0.63)	0.8 (0.72–0.87)	0.67 (0.6–0.74)	0.61 (0.57–0.65)	0.56 (0.51–0.61)	0.5 (0.46–0.54)	0.68 (0.63–0.72)

*MCI* mild cognitive impairment, *NC* normal cognition

^a^Source: [5]

^b^Source: [21]

^c^Source: [23]

T-tau and p-tau strongly correlated across the total group (*r* = .92, *p* < .001); however, when comparing classification of individuals based on t-tau and p-tau, concordance was somewhat lower (79%; Table 2). Applying mixture modelling in ADNI showed that similar to the ADC, a tetramodal distribution best fitted the CSF t-tau and p-tau data (log-likelihood ratio for 3 vs 4 distributions, for t-tau: 25.2 and for p-tau: 54.3, both with *p* < 0.05, no further improvement for 5 distributions: log-likelihood ratio for 5 vs 4 distributions, for t-tau: 11.2, and p-tau: 20.3, with *p* = 0.08 and *p* = 0.05, respectively). The tetramodal distribution yielded three different cut-points for t-tau measured with Luminex (95%CI)—54 (42–68), 95 (68–125) and 171 (146–263) pg/mL respectively (Fig. 1b), and for p-tau levels (95%CI)—23 (20–28), 46 (38–57) and 99 (74–124). Comparing the first cut-point of t-tau (54 pg/mL) with the cut-point of 93 pg/mL previously determined for ADNI [21], our new cut-point for t-tau resulted in higher sensitivity to detect clinical AD dementia versus controls, at the cost of lower specificity (Table 4). The first p-tau cut-point (23 pg/mL) was identical to the cut-point reported in the literature [21].

In ADNI, we further repeated analyses on the novel Elecsys data as an analytical validation, and again observed a tetramodal distribution for t-tau (log-likelihood ratio for 3 vs 4 distributions, for t-tau: 12.4, and p-tau: 19.6, both with *p* < 0.05, no further improvement for 5 distributions: log-likelihood ratio for 5 vs 4 distributions, for t-tau: 7.1, and p-tau 10.6, both *p* > 0.05). The tetramodal distribution yielded for t-tau the cut-points (95%CI)—192 (129–235), 312 (239–405) and 515 (417–679), and for p-tau (95%CI)—21 (17–23), 36 (28–43) and 55 (47–68) (Fig. 1c). For the first t-tau cut-point measured with Elecsys (192 pg/mL) compared to the cut-point of 300 pg/mL previously reported for ADNI [23], our new cut-point also yielded higher sensitivity but lower specificity for Elecsys t-tau (Table 4). For Elecsys p-tau, the first cut-point of 21 pg/mL was comparable to a previously reported cut-point of 24 pg/mL [23] and yielded similar sensitivity and lower specificity estimates (Table 4). In ADNI, 81% of the subjects were labelled identically using t-tau labels for Luminex and Elecsys, whereas between-platform correspondence for p-tau labelling was 60% (Table 2). For Luminex, the correlation between t-tau and p-tau was .67 (*p* < .001) and the correspondence of t-tau and p-tau labelling was 52%. For Elecsys, the correlation between t-tau and p-tau was .98 (*p* < .001), and the correspondence of subgroup labelling was 71%.

### Clinical and biological characteristics of tau subgroups

**Fig. 2:**
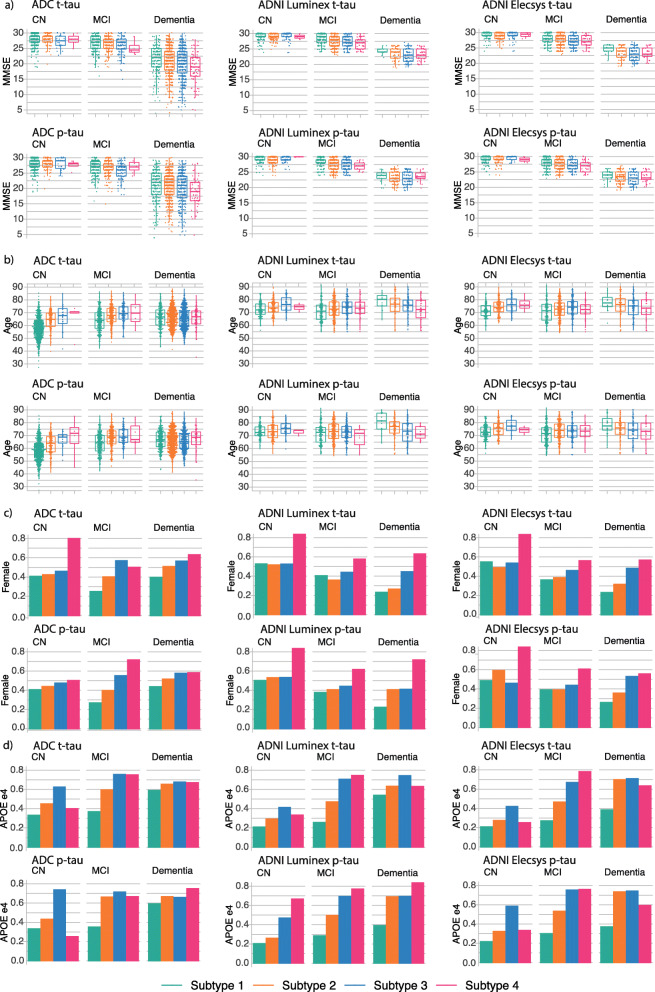
T-tau and p-tau subgroup comparisons within each cohort, stratified for cognitive state. Left, ADC; middle, ADNI Luminex; right, ADNI Elecsys. Comparisons for MMSE are shown in (**a**), for age in (**b**), for proportion female in (**c**) and for proportion of APOE-e4 carriers in (**d**). See Table 3 for statistical descriptions. NC, normal cognition; MCI, mild cognitive impairment; AD dementia, AD-type dementia

Gradually higher t- and p-tau subgroups in ADC were characterized by increasingly high prevalence of abnormal amyloid, with the highest two t-tau and p-tau groups consisting for more than 94% of amyloid abnormal participants. This relationship also held for lower t-tau values, with a higher prevalence of abnormal amyloid in the second subgroup than the lowest tau subgroup. T- and p-tau subgroups were also associated with cognitive state, with lower subgroups containing the highest proportion of cognitively normal participants, while highest subgroups contained more demented participants (Table 3). Therefore, we stratified subsequent comparisons between tau subgroups for cognitive state. Average MMSE was lower for higher tau subgroups, with the strongest effects observed in AD-type dementia (Fig. 2; Table 3). Tau subgroups also differed in demographic factors, including age (on average lower in the lowest tau subgroup in NC and MCI), sex (higher proportion of women in higher t-tau and p-tau subgroups), and APOE e4 carriership (higher prevalence in higher t-tau and p-tau subgroups) (Fig. 2; Table 3). The associations of higher t- and p-tau subgroups with amyloid, cognitive state, and demographic factors were mostly reproduced in ADNI.

### Rates of cognitive decline over time depend on tau subgroups

**Fig. 3:**
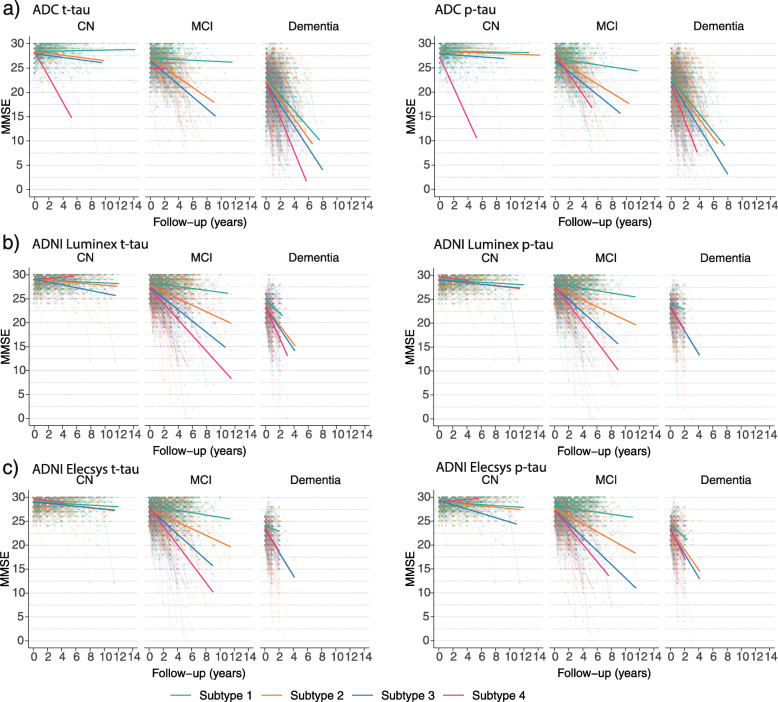
Comparison of annual MMSE decline for t-tau and p-tau subgroups, stratified for cognitive state. Left, t-tau; right, p-tau; top, ADC; middle, ADNI Luminex; bottom, ADNI Elecsys. See Table 5 for statistical descriptions. NC, normal cognition; MCI, mild cognitive impairment; AD dementia, AD-type dementia

We further studied whether subjects across tau subgroups differed in rates of cognitive decline, as measured with the MMSE stratified for cognitive state. In ADC, tau subgroups were not associated with cognitive decline in MCI or NC; however, in the dementia phase, higher tau subgroups were characterized by faster cognitive decline on MMSE (Fig. 3; Table 4). In ADNI, faster MMSE decline with higher tau subgroups in dementia was reproduced. While in ADC no association between tau subgroups and MMSE decline was found for participants with MCI, in ADNI, higher tau subgroups in MCI were associated with MMSE decline (Table 5).

**Table 5:** Tau subgroup comparisons on MMSE at first visit and annual change rates

				Subgroup effect	Baseline estimated marginal means ± SE of subgroup				*p* values of pairwise comparisons between subgroups						Time effect	Subgroup x Time	Estimated slopes ± SE of subgroup				*p* value of pairwise comparisons of slope differences between subgroups					
Bio-marker	Cognitive state	Cohort: platform	N per subgroup 1/2/3/4	*p* value	Subgroup 1	Subgroup 2	Subgroup 3	Subgroup 4	1 vs 2	1 vs 3	1 vs 4	2 vs 3	2 vs 4	3 vs 4	*p* value	*p* value	Subgroup 1	Subgroup 2	Subgroup 3	Subgroup 4	1 vs 2	1 vs 3	1 vs 4	2 vs 3	2 vs 4	3 vs 4
T-tau	NC	ADC: Innotest	240/73/13/4	0.428	28.4 ± 0.1	28 ± 0.2	27.3 ± 0.4	23.8 ± 0.9	6.45E−02	**1.07E**−**02**	**2.00E**−**07**	1.15E−01	**2.20E**−**06**	**2.54E**−**04**	**1.14E−07**	**1.34E−05**	0 ± 0.1	−0.2 ± 0.1 ^a^	−0.3 ± 0.2	−2.2 ± 0.5 ^c^	6.16E−02	1.51E−01	**2.65E**−**06**	7.06E−01	**1.97E**−**05**	**8.86E**−**05**
		ADNI: Luminex	151/151/60/2	0.231	28.8 ± 0.1	28.8 ± 0.1	28.6 ± 0.1	29.3 ± 0.6	8.90E−01	1.07E−01	5.10E−01	7.92E−02	5.25E−01	3.06E−01	3.12E−01	**3.23E−03**	0 ± 0	−0.1 ± 0 ^b^	− 0.3 ± 0 ^c^	0.1 ± 0.3	1.38E−01	**3.36E**−**04**	5.15E−01	**1.21E**−**02**	3.55E−01	1.36E−01
		ADNI: Elecsys	124/163/67/3	0.725	28.8 ± 0.1	28.8 ± 0.1	28.6 ± 0.1	28.7 ± 0.5	7.25E−01	1.58E−01	8.06E−01	2.20E−01	8.65E−01	8.82E−01	9.20E−02	1.15E−01	0 ± 0	−0.1 ± 0 ^b^	−0.2 ± 0 ^c^	0 ± 0.2	2.32E−01	**1.60E**−**02**	9.47E−01	1.20E−01	7.42E−01	4.69E−01
	MCI	ADC: Innotest	199/170/92/4	0.270	26.6 ± 0.2	25 ± 0.2	24.2 ± 0.3	24.2 ± 1.7	**7.98E**−**09**	**1.13E**−**11**	1.47E−01	**2.99E**−**02**	6.39E−01	9.71E−01	**2.05E−03**	**8.94E−13**	−0.2 ± 0.1 ^a^	−1.0 ± 0.1 ^c^	− 1.3 ± 0.1 ^c^	−0.6 ± 1	**2.48E−10**	**3.78E**−**11**	6.98E−01	1.35E−01	6.68E−01	5.13E−01
		ADNI: Luminex	162/237/145/56	**0.0002**	27.9 ± 0.2	26.4 ± 0.2	25.1 ± 0.2	24 ± 0.4	**1.36E**−**06**	**4.46E**−**16**	**4.12E**−**17**	**3.32E**−**05**	**3.80E**−**08**	**1.39E**−**02**	**1.74E−47**	**7.39E−17**	−0.2 ± 0.1 ^a^	−0.7 ± 0.1 ^c^	−1.2 ± 0.1 ^c^	− 1.6 ± 0.2 ^c^	**4.68E**−**05**	**3.56E**−**13**	**1.82E**−**14**	**7.80E**−**05**	**1.39E**−**07**	**1.69E**−**02**
		ADNI: Elecsys	145/247/154/41	**0.0001**	27.7 ± 0.2	26.7 ± 0.2	25.1 ± 0.2	23.7 ± 0.5	**9.96E**−**04**	**2.04E**−**14**	**4.56E**−**14**	**1.12E**−**07**	**3.71E**−**09**	**9.62E**−**03**	**7.02E−43**	**7.36E−16**	−0.2 ± 0.1 ^a^	− 0.6 ± 0.1 ^c^	−1.1 ± 0.1 ^c^	− 1.8 ± 0.2 ^c^	**5.03E**−**04**	**2.90E**−**11**	**3.83E**−**14**	**6.21E**−**05**	**5.28E**−**09**	**9.50E**−**04**
	AD dementia	ADC: Innotest	122/303/280/38	0.172	20.4 ± 0.4	19.7 ± 0.3	18.9 ± 0.3	17.1 ± 0.8	1.58E−01	**3.00E**−**03**	**1.58E**−**04**	**3.87E**−**02**	**1.32E**−**03**	**2.61E**−**02**	**1.72E−71**	**1.73E−04**	−1.7 ± 0.2 ^c^	−2.0 ± 0.1 ^c^	−2.3 ± 0.1 ^c^	− 3.5 ± 0.4 ^c^	1.49E−01	**1.55E**−**02**	**1.95E**−**05**	2.00E−01	**1.92E**−**04**	**1.69E**−**03**
		ADNI: Luminex	13/63/95/43	**0.031**	23.9 ± 0.9	22 ± 0.4	21.6 ± 0.3	20.8 ± 0.5	5.04E−02	**1.41E**−**02**	**2.35E**−**03**	4.16E−01	5.82E−02	1.77E−01	**3.33E−16**	1.29E−01	−1.5 ± 0.8 ^a^	−2.0 ± 0.4 ^c^	− 2.4 ± 0.3 ^c^	− 3.2 ± 0.5 ^c^	6.20E−01	3.06E−01	6.60E−02	3.67E−01	**3.53E**−**02**	1.34E−01
		ADNI: Elecsys	13/68/99/28	**0.008**	25 ± 0.9	21.8 ± 0.4	21.3 ± 0.3	21.3 ± 0.6	**1.00E**−**03**	**1.33E**−**04**	**6.41E**−**04**	3.69E−01	4.77E−01	9.33E−01	**8.53E−13**	**3.68E−02**	−0.3 ± 0.8	−2.2 ± 0.4 ^c^	− 2.4 ± 0.3 ^c^	− 3.0 ± 0.6 ^c^	**2.48E**−**02**	**9.79E**−**03**	**4.88E**−**03**	6.24E−01	2.33E−01	3.65E−01
P-tau	NC	ADC: Innotest	237/76/15/2	0.120	28.4 ± 0.1	28.2 ± 0.2	27.5 ± 0.4	22.2 ± 1	2.84E−01	**2.48E**−**02**	**1.10E**−**08**	1.03E−01	**3.91E**−**08**	**3.65E**−**06**	**2.80E−07**	**3.36E−05**	−0.1 ± 0.1	−0.1 ± 0.1	−0.2 ± 0.2	−2.5 ± 0.5 ^c^	3.79E−01	4.50E−01	**2.05E**−**06**	7.62E−01	**5.29E**−**06**	**2.55E**−**05**
		ADNI: Luminex	137/164/59/3	0.883	28.9 ± 0.1	28.7 ± 0.1	28.7 ± 0.1	29.1 ± 0.8	6.64E−02	1.13E−01	7.68E−01	7.89E−01	5.74E−01	5.43E−01	2.10E−01	1.35E−01	0 ± 0	−0.1 ± 0	−0.2 ± 0.1 ^b^	−0.1 ± 0.4	5.87E−02	**4.56E**−**02**	8.32E−01	4.88E−01	9.82E−01	8.85E−01
		ADNI: Elecsys	206/124/24/2	0.367	28.8 ± 0.1	28.7 ± 0.1	28.5 ± 0.2	29.2 ± 0.7	2.81E−01	1.70E−01	5.56E−01	4.27E−01	4.39E−01	3.15E−01	1.24E−01	**8.11E**−**03**	−1.3 ± 0.6 ^a^	−2.1 ± 0.3 ^c^	−2.7 ± 0.4 ^c^	−3.0 ± 0.6 ^c^	1.74E−01	**3.60E**−**02**	**3.29E**−**02**	2.52E−01	1.83E−01	5.96E−01
	MCI	ADC: Innotest	208/173/79/5	0.575	26.3 ± 0.2	25.1 ± 0.2	24.3 ± 0.3	23.8 ± 1.4	**6.13E**−**05**	**7.46E**−**08**	**7.69E**−**02**	**2.19E**−**02**	3.41E−01	7.18E−01	**2.94E−09**	**5.39E−08**	−0.4 ± 0.1 ^c^	−0.9 ± 0.1 ^c^	−1.2 ± 0.1 ^c^	−2.0 ± 0.7 ^b^	**1.32E**−**05**	**1.08E**−**07**	**2.23E**−**02**	6.44E−02	1.32E−01	2.75E−01
		ADNI: Luminex	164/259/161/12	**0.009**	27.7 ± 0.2	26.3 ± 0.2	25 ± 0.2	23.8 ± 0.8	**1.03E**−**06**	**8.81E**−**17**	**8.02E**−**06**	**1.20E**−**05**	**4.47E**−**03**	1.95E−01	**2.19E−27**	**3.33E−16**	−0.2 ± 0.1 ^a^	−0.7 ± 0.1 ^c^	−1.3 ± 0.1 ^c^	−2.0 ± 0.3 ^c^	**1.04E**−**04**	**2.19E**−**16**	**3.76E**−**07**	**1.31E**−**07**	**1.50E**−**04**	6.60E−02
		ADNI: Elecsys	228/224/103/32	**0.011**	27.6 ± 0.2	26.1 ± 0.2	24.6 ± 0.3	23.7 ± 0.5	**6.25E**−**08**	**3.34E**−**18**	**1.74E**−**12**	**8.99E**−**06**	**1.14E**−**05**	1.32E−01	**1.76E−46**	**5.50E−19**	−0.2 ± 0.1 ^b^	−0.8 ± 0.1 ^b^	−1.4 ± 0.1 ^c^	−1.8 ± 0.2 ^c^	1.74E−01	**3.60E**−**02**	**3.29E**−**02**	2.52E−01	1.83E−01	5.96E−01
	AD dementia	ADC: Innotest	149/348/216/30	0.250	20.2 ± 0.4	19.4 ± 0.2	19 ± 0.3	16.3 ± 0.9	**7.19E**−**02**	**1.25E**−**02**	**4.35E**−**05**	2.97E−01	**6.82E**−**04**	**4.03E**−**03**	**2.75E−65**	**9.95E−05**	−1.8 ± 0.2 ^c^	−2 ± 0.1 ^c^	−2.4 ± 0.1 ^c^	−3.7 ± 0.4 ^c^	3.14E−01	**5.30E**−**03**	**7.50E**−**05**	**2.36E**−**02**	**2.48E**−**04**	**6.23E**−**03**
		ADNI: Luminex	17/98/83/14	0.483	23.7 ± 0.8	21.5 ± 0.3	21.5 ± 0.4	21.8 ± 0.9	**1.21E**−**02**	**1.30E**−**02**	1.20E−01	9.54E−01	7.47E−01	7.26E−01	**1.62E−10**	9.00E−02	−0.7 ± 0.7	−2.5 ± 0.3 ^c^	−2.6 ± 0.3 ^c^	−2.1 ± 0.8 ^a^	**2.00E**−**02**	**1.33E**−**02**	2.06E−01	7.23E−01	6.68E−01	5.53E−01
		ADNI: Elecsys	26/91/66/25	0.118	23.4 ± 0.7	21.8 ± 0.3	21.2 ± 0.4	21.2 ± 0.7	**3.21E**−**02**	**4.37E**−**03**	**1.91E**−**02**	2.39E−01	4.10E−01	9.88E−01	**1.88E−18**	**9.79E**−**02**	−1.3 ± 0.6 ^a^	−2.1 ± 0.3 ^c^	−2.7 ± 0.4 ^c^	−3.0 ± 0.6 ^c^	1.74E−01	**3.60E**−**02**	**3.29E**−**02**	2.52E−01	1.83E−01	5.96E−01

All analyses were stratified for cognitive state and adjusted for age, sex and level of education. Slope that differs from 0 is indicated with ^a^ when *p* < .05, ^b^ when *p* < .01 or ^c^ when *p* < .001

*NC* cognitively normal, *MCI* mild cognitive impairment, *AD* Alzheimer’s disease

**Fig. 4:**
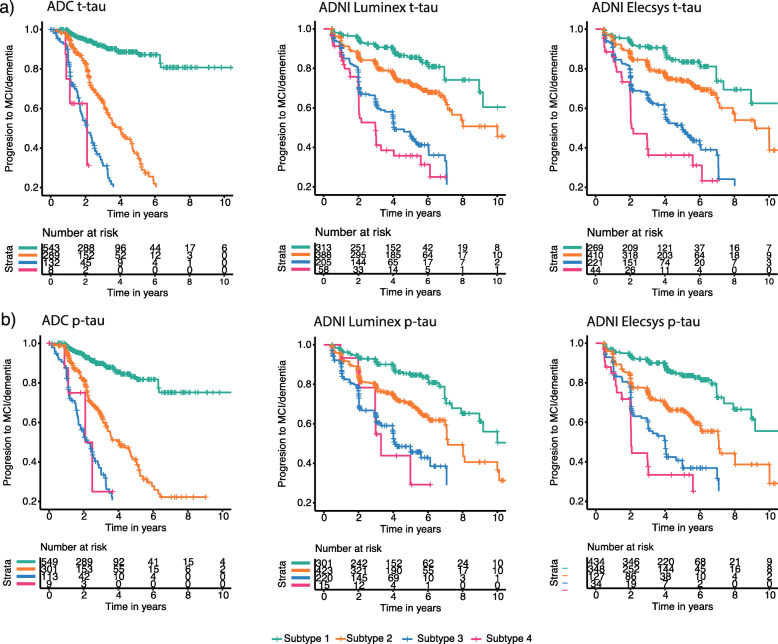
Proportional Hazard curves for progression to dementia in initially non-demented individuals in different tau subgroups. T-tau subgroups are shown in (**a**) and p-tau subgroups in (**b)**; left, ADC; middle, ADNI Luminex; right, ADNI Elecsys. See Table 6 for statistical descriptions

Next, we tested for individuals without dementia (i.e. NC and MCI) whether tau-subgroups differed in terms of progression to MCI or AD-type dementia. In the ADC, 46/381 (12%) of NC patients showed clinical progression either to MCI (*n* = 39) within 2.3 ± 1.6 years or to AD-type dementia (*n* = 16) in 4.5 ± 4.0 years, and 178/591 (30%) of MCI patients progressed to AD-type dementia in 2.4 ± 1.6 years. Across the total group of non-demented subjects, hazard ratios (HRs) increased with increasing tau or p-tau subgroups compared to the lowest tau or p-tau subgroups (Fig. 4; Table 6). Repeating analyses including covariates sex, age and education level (model 2), amyloid status (model 3), baseline cognitive state (model 4) and APOE-e4 carriership (model 5) generated largely similar results for t-tau subgroups, although HRs were somewhat attenuated. Results were largely consistent for ADNI albeit with somewhat lower HR values (Table 6), where 65/371 (17.5%) NC showed clinical progression either to MCI (*n* = 47) within 3 ± 9 years or to AD-type dementia (*n* = 18) in 8 ± 3 years, and 212/622 (34%) MCI individuals to AD-type dementia in 4.1 ± 2.3 years. Of note is that in ADNI, individuals in the second Luminex t-tau subgroup had levels below the official cut-point defined by ADNI (i.e. 93 pg/ml [21]) and still showed higher HRs for progression to AD-type dementia compared to the first tau subgroup (HR (95%CI) = 2.1 (1.4, 3.0), *p* < .001).

**Table 6:** Cox proportional hazard models for time to progression in individuals with NC or MCI

		Model 1: no covariates		Model 2: + sex, age, education		Model 3: model 2 + amyloid status		Model 4: model 3 + diagnosis		Model 5: model 4+ APOE-e4 carriership	
		HR (95%CI)	*p*	HR (95%CI) sex, age, edu	p	HR (95%CI) sex, age, edu, abeta	*p*	HR (95%CI) sex, age, edu, abeta, diagnosis	*p*	HR (95%CI) sex, age, edu, abeta, diagnosis, APOE-e4	*p*
T-tau subgroups											
Subgroup 1	All	Reference									
Subgroup 2	ADC: Innotest	6.3 (4.3, 9.2)	5.79E−21	6 (3.9, 9.2)	1.32E−16	3.4 (2.2, 5.3)	5.96E−08	3.4 (2.1, 5.3)	1.08E−07	3.3 (2.1, 5.3)	4.31E−07
	ADNI: Luminex	2.1 (1.4, 3)	8.76E−05	2 (1.4, 3)	1.58E−04	1.7 (1.2, 2.5)	5.84E−03	1.6 (1.1, 2.4)	1.24E−02	1.5 (1, 2.2)	2.98E−02
	ADNI: Elecsys	1.9 (1.3, 2.8)	9.27E−04	1.8 (1.2, 2.7)	2.44E−03	1.8 (1.2, 2.7)	2.56E−03	1.8 (1.2, 2.6)	3.71E−03	1.7 (1.1, 2.5)	9.74E−03
Subgroup 3	ADC: Innotest	14.6 (9.8, 21.8)	6.14E−39	13.2 (8.3, 20.8)	3.31E−28	6.2 (3.8, 10)	1.96E−13	6.0 (3.7, 9.8)	6.96E−13	6.1 (3.7, 10.1)	1.86E−12
	ADNI: Luminex	5.2 (3.6, 7.6)	1.31E−18	4.9 (3.4, 7.2)	3.53E−17	2.8 (1.9, 4.1)	2.86E−07	2.6 (1.8, 3.9)	1.57E−06	2.4 (1.6, 3.6)	1.06E−05
	ADNI: Elecsys	4.4 (3, 6.4)	6.15E−14	4 (2.7, 5.9)	2.05E−12	3.1 (2.1, 4.5)	1.88E−08	2.8 (1.9, 4.2)	1.91E−07	2.7 (1.8, 4)	1.60E−06
Subgroup 4	ADC: Innotest	21.3 (7.5, 60.6)	9.81E−09	15 (4.4, 50.9)	1.43E−05	6.5 (1.9, 22.3)	2.93E−03	6.6 (1.9, 22.7)	2.70E−03	6.4 (1.9, 22.2)	3.30E−03
	ADNI: Luminex	6.7 (4.2, 10.7)	1.01E−15	6.6 (4.1, 10.6)	3.89E−15	3.4 (2.1, 5.6)	8.31E−07	2.8 (1.7, 4.5)	6.50E−05	2.6 (1.5, 4.2)	2.63E−04
	ADNI: Elecsys	7.1 (4.3, 11.7)	2.96E−14	7.2 (4.3, 12)	2.37E−14	4.6 (2.7, 7.7)	6.71E−09	3.9 (2.3, 6.6)	2.99E−07	3.5 (2.1, 6.1)	3.59E−06
P-tau subgroups											
Subgroup 1	All	Reference									
Subgroup 2	ADC: Innotest	4.4 (3.1, 6.2)	4.13E−17	3.9 (2.7, 5.7)	2.35E−12	2.1 (1.4, 3.2)	1.71E−04	2.1 (1.4, 3.1)	2.28E−04	2.1 (1.4, 3.2)	4.88E−04
	ADNI: Luminex	2.2 (1.5, 3)	7.78E−06	2.1 (1.5, 3)	1.38E−05	1.5 (1.0, 2.1)	3.30E−02	1.4 (1.0, 2.0)	5.85E−02	1.4 (1, 2)	7.19E−02
	ADNI: Elecsys	2.6 (1.9, 3.6)	9.65E−10	2.6 (1.9, 3.5)	3.83E−09	2.2 (1.6, 3.0)	1.73E−06	2.0 (1.5, 2.8)	1.22E−05	2 (1.4, 2.7)	4.34E−05
Subgroup 3	ADC: Innotest	10.2 (7, 14.8)	1.48E−33	8.4 (5.6, 12.8)	1.10E−23	3.7 (2.4, 5.8)	6.58E−09	3.6 (2.3, 5.6)	2.03E−08	3.5 (2.2, 5.6)	1.02E−07
	ADNI: Luminex	4.4 (3.1, 6.3)	1.81E−16	4.3 (3.0, 6.2)	4.37E−16	2.3 (1.6, 3.3)	1.80E−05	2.2 (1.5, 3.2)	7.21E−05	2 (1.4, 3)	2.88E−04
	ADNI: Elecsys	5.0 (3.6, 7.1)	7.09E−20	4.7 (3.3, 6.7)	4.35E−18	3.0 (2.1, 4.3)	4.19E−09	2.7 (1.9, 3.9)	1.32E−07	2.6 (1.8, 3.7)	8.22E−07
Subgroup 4	ADC: Innotest	9.5 (3.4, 26.5)	1.69E−05	4.6 (1.4, 15.5)	1.28E−02	2.1 (0.6, 7.2)	2.19E−01	2.1 (0.6, 7.1)	2.29E−01	4 (1.2, 13.8)	2.72E−02
	ADNI: Luminex	5.0 (2.4, 10.6)	2.83E−05	5.8 (2.7, 12.4)	5.32E−06	2.5 (1.2, 5.5)	1.76E−02	2.3 (1.0, 4.9)	3.81E−02	2.1 (0.9, 4.5)	6.85E−02
	ADNI: Elecsys	7.7 (4.7, 12.6)	3.47E−16	8.2 (4.9, 13.4)	1.24E−16	4.7 (2.8, 7.7)	3.52E−09	3.9 (2.3, 6.6)	2.51E−07	3.6 (2.1, 6.1)	2.11E−06
Amyloid status											
Amyloid abnormal	ADC: Innotest (< 813 pg/ml)^1^	10.8 (7.3, 15.9)	8.71E−33	8.9 (5.9, 13.4)	6.65E−25	n.t.		7.8 (5.0, 12.0)	1.88E−20	7.2 (4.5, 11.5)	5.27E−17
	ADNI: Luminex (< 192 pg/ml)^2^	5.2 (3.8, 7.0)	2.29E−25	5.0 (3.6, 6.8)	4.91E−24	n.t.		4.4 (3.2, 6.0)	1.79E−20	3.8 (2.7, 5.3)	4.16E−15
	ADNI: Elecsys (< 880 pg/ml)^3^	4.2 (3.2, 5.5)	1.60E−26	4.1 (3.1, 5.3)	8.27E−25	n.t.		3.5 (2.7, 4.6)	1.30E−19	3 (2.2, 4)	2.51E−13
Continuous predictors											
Continuous ab1-42 (*z* score; HR per SD)	ADC: Innotest	0.3 (0.2, 0.4)	3.31E−36	0.3 (0.3, 0.4)	8.16E−27	0.6 (0.4, 0.8)	2.84E−03	0.6 (0.4,0.9)	4.17E−03	0.6 (0.4, 0.8)	4.75E−03
	ADNI: Luminex	0.5 (0.4, 0.5)	1.71E−30	0.5 (0.4, 0.5)	6.62E−23	0.6 (0.5, 0.8)	7.12E−05	0.7 (0.5, 0.9)	2.19E−03	0.7 (0.6, 0.9)	1.79E−02
	ADNI: Elecsys	0.4 (0.3, 0.5)	2.24E−23	0.4 (0.3, 0.5)	7.05E−22	0.6 (0.4, 0.8)	1.14E−04	0.6 (0.5,0.8)	1.56E−04	0.6 (0.5, 0.8)	5.41E−04
Continuous t-tau (*z* score; HR per SD)	ADC: Innotest	1.6 (1.5, 1.7)	7.67E−58	1.9 (1.7, 2.1)	3.60E−32	1.5 (1.3, 1.7)	2.42E−11	1.5 (1.3, 1.7)	4.57E−11	1.6 (1.4, 1.8)	3.46E−11
	ADNI: Luminex	1.7 (1.6, 1.9)	7.95E−32	1.7 (1.6, 1.9)	5.61E−31	1.4 (1.3, 1.6)	1.80E−11	1.4 (1.2, 1.5)	1.15E−08	1.3 (1.2, 1.5)	1.69E−07
	ADNI: Elecsys	1.6 (1.5, 1.8)	8.36E−27	1.7 (1.5, 1.8)	1.39E−25	1.4 (1.3, 1.6)	2.41E−13	1.4 (1.3, 1.5)	1.51E−10	1.4 (1.2, 1.5)	4.90E−09
Continuous p-tau (*z* score; HR per SD)	ADC: Innotest	1.8 (1.7, 2.0)	2.92E−42	1.7 (1.5, 1.9)	3.31E−22	1.4 (1.2, 1.6)	1.11E−07	1.4 (1.2, 1.6)	3.56E−07	1.5 (1.3, 1.7)	3.03E−08
	ADNI: Luminex	1.5 (1.4, 1.6)	5.58E−24	1.6 (1.4, 1.7)	1.75E−25	1.3 (1.2, 1.5)	1.67E−08	1.3 (1.2, 1.4)	2.46E−07	1.3 (1.1, 1.4)	4.28E−06
	ADNI: Elecsys	1.7 (1.5, 1.8)	3.36E−31	1.7 (1.5, 1.9)	1.06E−29	1.5 (1.3, 1.6)	1.72E−13	1.4 (1.3, 1.5)	9.62E−11	1.4 (1.2, 1.5)	3.05E−09

*N.t.* not tested

^1^Source: Tijms BM et al., Clinical Chemistry. 2018;64(3):576–585

^2^Source: [21]

^3^Source: Hansson O et al., Alzheimer’s & Dementia. 2018;14(11):1470–1481

### Longitudinal changes in tau concentrations in ADNI

**Fig. 5:**
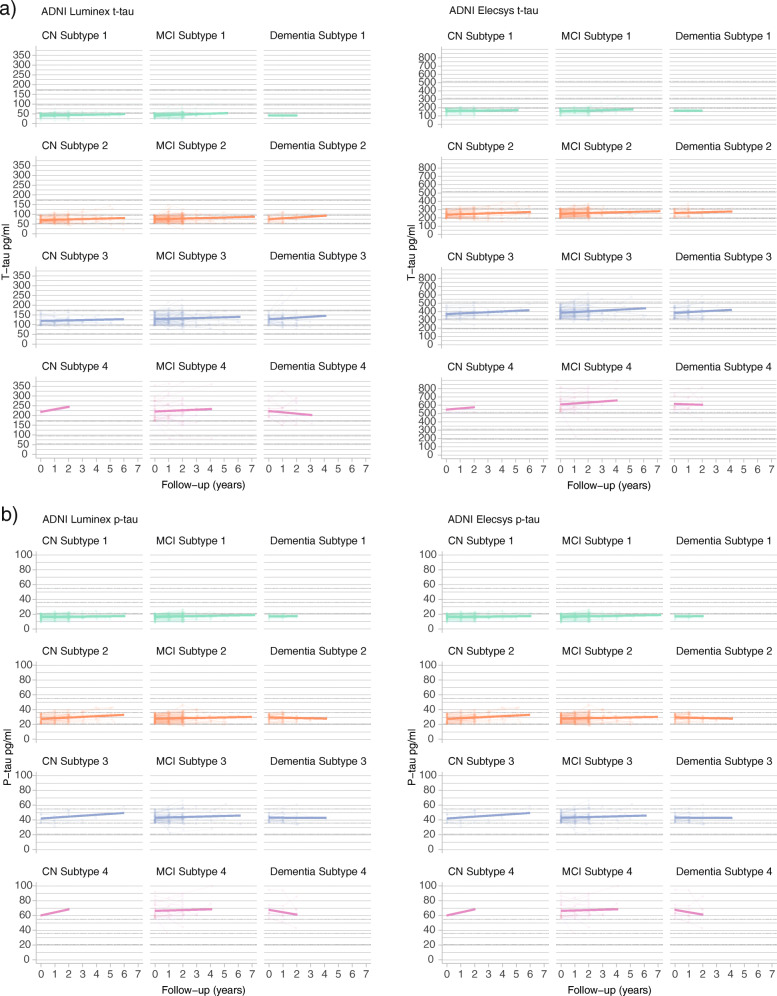
Changes over time in t-tau and p-tau levels, stratified for tau subgroup and cognitive state. Changes in t-tau levels are shown in (**a**) and in p-tau levels in (**b**). Left, ADNI Luminex; right, ADNI Elecsys. See Table 7 for statistical descriptions. NC, normal cognition; MCI, mild cognitive impairment; AD dementia, AD-type dementia

Examining transitions over time to higher tau groups in ADNI, we observed that the majority of individuals for both Luminex and Elecsys t-tau subgroups remained in the same subgroup as first measured (Luminex: 472 (76% of 619); Elecsys: 443 (76% of 586); Table 7; see Table 8 and Fig. 5 for continuous results). Of individuals who changed, the majority shifted to one tau group higher (Table 8).

**Table 7:** Frequencies of individuals remaining or changing subgroup over time from baseline (rows)

Biomarker: Platform		Subgroup at last measurement			
T-tau: Luminex	Baseline subgroup	1	2	3	4
	1	110	35	1	0
	2	22	188	42	0
	3	0	18	139	15
	4	0	1	13	35
T-tau: Elecsys	Baseline subgroup	1	2	3	4
	1	92	25	1	0
	2	11	206	36	0
	3	0	17	147	13
	4	0	1	4	33
P-tau: Luminex	Baseline subgroup	1	2	3	4
	1	103	63	11	0
	2	24	164	91	5
	3	2	23	100	13
	4	0	1	6	6
P-tau: Elecsys	Baseline subgroup	1	2	3	4
	1	185	33	0	0
	2	9	197	25	0
	3	0	14	75	12
	4	0	0	4	33

**Table 8:** T-tau and p-tau subgroup comparisons on annual change in CSF t-tau and p-tau values

	Effect time			Interaction subgroup x time		Annual change (se) for each subgroup				*p* values pairwise comparisons in slope differences between subgroups						Baseline effect tau group		Baseline estimated marginal means (SE) for each subgroup				*p* values pairwise comparisons in baseline estimates between subgroups					
Biomarker: platform	*F*	*p* value		*F*	*p* value	Subgroup 1	Subgroup 2	Subgroup 3	Subgroup 4	1 vs 2	1 vs 3	1 vs 4	2 vs 3	2 vs 4	3 vs 4	*F*	*p* value	Subgroup 1	Subgroup 2	Subgroup 3	Subgroup 4	1 vs 2	1 vs 3	1 vs 4	2 vs 3	2 vs 4	3 vs 4
T-tau: Luminex	14	1.74E−04		0.2	8.96E−01	1.72 (0.75) ^a^	2.03 (0.57) ^c^	2.2 (0.71) ^b^	1.08 (1.44)	7.45E−01	6.43E−01	6.94E−01	8.50E−01	5.41E−01	4.86E−01	1318	7.48E−273	44.6 (1.89)	76.4 (1.43)	129.3 (1.73)	221.9 (3.24)	1.65E−36	1.83E−137	6.43E−206	1.68E−87	3.96E−177	3.88E−96
T-tau: Elecsys	37	2.55E−09		2.5	5.98E−02	3.34 (1.68) ^a^	4.8 (1.16) ^c^	8.71 (1.46) ^c^	8.36 (3.31) ^a^	4.74E−01	**1.62E**−**02**	1.77E−01	**3.67E**−**02**	3.11E−01	9.23E−01	1330	1.30E−261	162.4 (4.49)	254.1 (3.05)	392.8 (3.64)	616.8 (7.86)	7.82E−53	1.58E−168	1.28E−215	7.20E−117	1.89E−184	4.47E−99
P-tau: Luminex	2	1.63E−01		15	**2.93E**−**09**	3.17 (0.72) ^c^	4.67 (0.6) ^c^	2.89 (0.88) ^b^	−15.23 (2.95) ^c^	1.11E−01	8.07E−01	**2.58E**−**09**	9.58E−02	**8.98E**−**11**	**6.95E**−**09**	808	6.11E−222	21.8 (1.06)	39.1 (0.84)	65.5 (1.22)	109.5 (3.99)	9.63E−33	2.98E−101	1.14E−73	5.97E−56	9.88E−54	7.98E−24
P-tau: Elecsys	0.005	2.85E−02		1.5	2.13E−01	0.26 (0.13)^a^	0.48 (0.13) ^c^	0.49 (0.21) ^a^	−0.22 (0.37)	2.12E−01	3.28E−01	2.25E−01	9.68E−01	7.35E−02	9.28E−02	1456	3.10E−270	16.8 (0.36)	28.4 (0.34)	43.4 (0.52)	66.0 (0.90)	1.96E−181	7.62E−221	7.62E−221	1.64E−90	1.76E−167	6.98E−78

CSF t- and p-tau values are in pg/ml. Baseline effects are reported in the last columns. Bold font highlights significant effects. Slope that differs from 0 is indicated with ^a^ when *p* < .05, ^b^ when *p* < .01 or ^c^ when *p* < .001

*SE* standard error

### Comparison with tau PET in ADNI

**Fig. 6:**
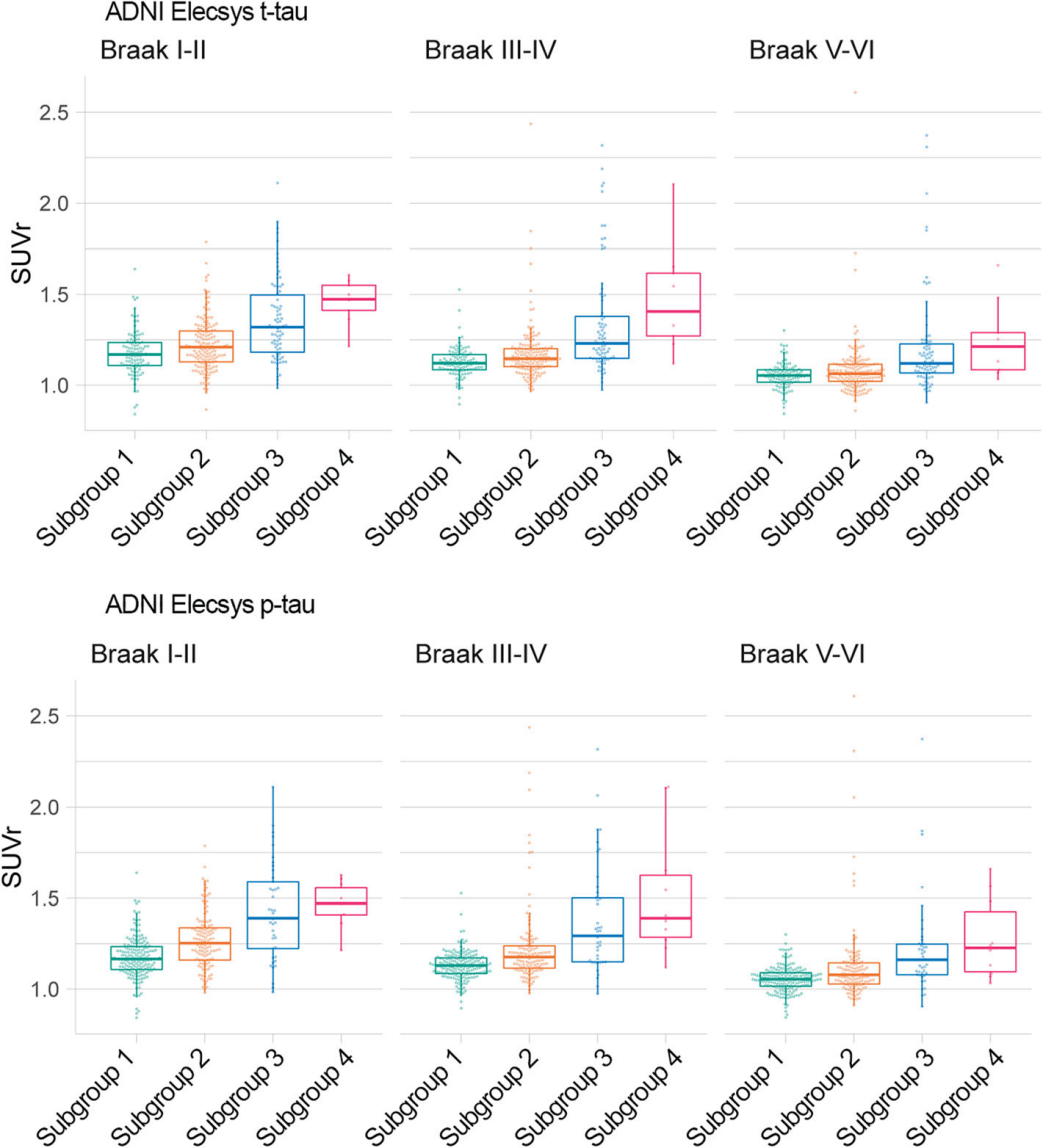
Comparison of t-tau and p-tau subgroups in tau PET uptake according to Braak stages. Tau PET uptake for t-tau subgroups are shown in (**a**) and for p-tau subgroups in (**b**). Left, ADNI Luminex; right, ADNI Elecsys. See Table 9 for statistical descriptions. SUVr, standardized uptake value ratio

Finally, we compared CSF tau subgroups on tau PET uptake values available for 345 individuals (235 NC; 93 MCI; 28 dementia; of note, these included *n* = 232 new CSF observations not included in mixture analyses). Figure 6 shows that tau PET uptake increased with higher t-tau and p-tau subgroups. For all Braak regions, the uptake of the highest two tau subgroups was significantly higher than the lowest two (or three) subgroups (Table 9). The second lowest t-tau subgroup also showed higher average tau uptake in Braak I/II brain areas compared to subgroup 1, and the second lowest p-tau subgroup in addition also to Braak III/IV and V/VI compared to subgroup 1.

**Table 9:** T-tau and p-tau subgroup comparison on tau PET uptake

		T-tau subgroup effect		T-tau subgroup estimated marginal means (SE)				T-tau subgroup pairwise comparisons *p* value					
Tau PET SUVr	Subgroup	*F*	*p* value	Subgroup 1	Subgroup 2	Subgroup 3	Subgroup 4	1 vs 2	1 vs 3	1 vs 4	2 vs 3	2 vs 4	3 vs 4
Braak I/II	T-tau	28	3.69E**−**16	1.18 (0.016)	1.22 (0.013)	1.36 (0.018)	1.51 (0.052)	**2.91E−02**	**1.91E−12**	**1.65E−09**	**8.36E−09**	**1.03E−07**	**4.78E−03**
	P-tau	37.6	5.14E**−**21	1.18 (0.012)	1.27 (0.014)	1.43 (0.024)	1.47 (0.049)	**1.02E−06**	**1.97E−18**	**1.95E−08**	**3.40E−08**	**1.22E−04**	4.67E**−**01
Braak III/IV	T-tau	30	2.64E**−**17	1.13 (0.019)	1.17 (0.016)	1.34 (0.022)	1.56 (0.061)	5.19E**−**02	**2.98E−12**	**6.58E−11**	**3.73E−09**	**3.08E−09**	**7.24E−04**
	P-tau	31	1.04E**−**17	1.13 (0.014)	1.23 (0.017)	1.38 (0.029)	1.51 (0.059)	**1.79E−05**	**1.50E−13**	**7.23E−10**	**7.87E−06**	**3.88E−06**	**4.08E−02**
Braak V/VI	T-tau	16	7.49E**−**10	1.05 (0.018)	1.09 (0.015)	1.21 (0.021)	1.32 (0.058)	1.50E**−**01	**3.80E−08**	**1.01E−05**	**2.69E−06**	**8.12E−05**	5.80E−02
	P-tau	14.2	1.04E**−**08	1.06 (0.014)	1.13 (0.016)	1.22 (0.028)	1.27 (0.056)	**4.88E−04**	**1.21E−07**	**1.97E−04**	**4.22E−03**	**1.49E−02**	4.21E**−**01

Bold font indicates significant group difference

## Discussion

In this study, we used Gaussian mixture modelling to determine unbiased cut-points for CSF tau levels. We identified three cut-points resulting in four different distributions, and the cut-point between the lowest two subgroups corresponded closely to an existing clinically defined cut-point [21]. Furthermore, two additional tau groups with highest t- and p-tau levels were discovered in the data. We similarly observed four distributions in the independent ADNI cohort, and despite differences between ADC and ADNI in cohort composition, tau subgroups showed similar clinical and biological characteristics in both study cohorts. These findings suggest that t-tau and p-tau levels may not necessarily reflect disease stage, but possibly different biological subtypes of AD.

Tau is an intracellular protein playing an important role in microtubule assembly and stabilization in axons [24]. Hyperphosphorylation disturbs its function, resulting in the formation of aggregates or neurofibrillary tangles, which is one of the hallmarks of AD pathology. Still, the precise factors influencing t- and p-tau CSF levels remain unclear. Measures correlated highly, and even though subgroup labelling showed moderate concordance, t-tau and p-tau subgroups showed similar differences in tau PET uptake. Previous studies comparing CSF tau measures with tau PET have been inconsistent [25–27]. Together with our results, it remains unclear whether CSF t-tau and p-tau reflect similar or different aspects of neuronal injury. Higher levels of t- and p-tau might result from passive release into extracellular space due to neuronal death which increases with worse disease severity. However, tau is also actively secreted by neurons as part of normal physiology [28] and can increase in the presence of amyloid pathology [29]. The majority of individuals remained in their t-tau subgroup over time, suggesting that at least part of their levels do not depend on disease stage, but perhaps reflect other biological aspects. The relative lack of change over time in tau levels within individuals seems at odds with the idea that tau increases with worsening cognition. Previous longitudinal CSF studies have reported conflicting results, observing increases in middle-age individuals with normal cognition during a follow-up period of 6 years [30], but also a lack of change in individuals with normal cognition, MCI and AD over a median follow-up of 2 years [31, 32]. This literature together with our observations suggests that increases over time in t-tau levels in CSF are slow, and follow-up times longer than 2–3 years might be necessary for participants to change subgroups.

One of the challenges in biomarkers research is how to define the cut-point between normal and abnormal levels. Pathology is the gold standard, but is also the end stage of the disease and difficult to obtain for large sample sizes. The cut-point for Luminex p-tau in ADNI was originally based on pathology [21], and we observed the same cut-off for the lowest p-tau subgroup (23 pg/ml). However, for t-tau, we observed a lower cut-off that was still related to increased risk for disease progression. A recent study defined cut-points for t- and p-tau measured with Elecsys (t-tau 300 pg/ml and p-tau 27 pg/ml) in ADNI based on their association with clinical progression in MCI patients [23]. We expand upon previous studies [6, 11, 33, 34] by identifying additional cut-points that may have practical use for more specific prognoses to individual patients or in trial design: we identified lower cut-points than defined in the literature (resp. 193 and 22 pg/ml for t- and p-tau, respectively) that were already associated with increased risk for clinical progression, and also showed for the higher cut-points, that the corresponding subgroups were associated with gradually increasing hazard ratios and steeper decline on the MMSE.

The notion that higher tau subgroups also included non-demented individuals, and that higher tau levels were associated with faster cognitive decline, regardless of disease stage, suggests that tau subgroups may reflect differences in underlying biological processes, rather than disease severity per se. This is supported by the observation that higher tau subgroups showed increasing proportions of APOE e4 carriers, the strongest genetic risk factor for AD [35]. Previous studies have also reported higher levels of tau in APOE-e4 carriers, also in predementia stages [36]; however, also see [37] where tau levels were similar between carriers and non-carriers. Other genetic risk factors may contribute to differences in tau levels as well, as another study reported that a polygenic risk score, including SNPs with moderate strength to detect AD, was strongly related to t-tau and p-tau levels, also after correcting for APOE [38]. This suggests that multiple genetic risk factors may explain variability between individual tau levels. More studies with large sample sizes are needed to further investigate these biological factors associated with tau levels in CSF. Also, future studies should further investigate the longitudinal relationship of these tau subtypes with concurrent other biological measures that deteriorate during the AD process, such as synaptic markers in CSF or on PET, and cognitive data, to better understand differences in clinical progression amongst tau subtypes.

### Limitations

A potential limitation of our study is that although we used large clinical cohorts, the number of subjects in some subgroups and subanalyses was small: this was especially the case in the highest tau subgroup, as well as in tau PET analyses. The small size of the highest tau subgroup means that there is more uncertainty in the association of this subgroup with clinical characteristics. Therefore, the results regarding the highest tau subgroup and the tau PET analyses should be interpreted with caution, and if possible repeated in future studies in even larger cohorts. Furthermore, we used Gaussian mixtures as a data-driven approach to study potential subgroups in tau levels as a first step, it is possible that more complex models may improve the fit of tau levels distributions, which should be addressed in future studies. Also, we determined cut-points here as the intersections of the probability distributions of the normal mixtures, which may not be ideal in all settings. For example, in studies where minimizing misclassification costs is desired, e.g. in clinical trial design, it may be useful to choose cut-points so that misclassification is minimized of individuals with high tau as falling in the lowest tau group, to ensure that as many individuals with potentially fast progression are included in the trial. Future studies could test the efficacy of the data-driven cut-points in those settings. Strengths of the study are that we used a large cohort, and we validated the mixture modelling results in another independent cohort with two different analysis platforms for CSF tau, and both cohorts had detailed information of the characteristics of the study populations, including cognitive measures, follow-up data on clinical progression and information on APOE genotype available.

## Conclusions

In conclusion, our studies suggest that abnormal levels of CSF t-tau and p-tau may convey different biological aspects in AD, which might be in part driven by genetic factors such as different APOE genotypes. The data-driven cut-points we found may aid daily practice in prognosis of patients and may aid trial design by allowing stratification of individuals according to their risk of clinical progression.

## Supplementary information

**Additional file 1.**

**Additional file 2.**

## Data Availability

The ADNI dataset analysed during the current study is available in the ADNI repository, www.adni-info.org. For ADC, the data that support the findings of this study are available from the corresponding author upon reasonable request.

## Abbreviations

Aβ42: Amyloid-β 1-42
AD: Alzheimer’s disease
ADC: Amsterdam Dementia Cohort
ADNI: Alzheimer’s disease Neuroimaging Initiative
APOE: Apolipoprotein E
CI: Confidence interval
CSF: Cerebrospinal fluid
HR: Hazard ratio
NC: Normal cognition
MCI: Mild cognitive impairment
MMSE: Mini-Mental State Examination
MRI: Magnetic resonance imaging
PET: Positron emission tomography
p-tau-181: Tau phosphorylated at threonine 181
t-tau: Total tau

## References

[1] Olsson B, Lautner R, Andreasson U, Öhrfelt A, Portelius E, Bjerke M (2016). CSF and blood biomarkers for the diagnosis of Alzheimer’s disease: a systematic review and meta-analysis. Lancet Neurol..

[2] McKhann GM, Knopman DS, Chertkow H, Hyman BT, Jack CR, Kawas CH (2011). The diagnosis of dementia due to Alzheimer’s disease: recommendations from the National Institute on Aging-Alzheimer’s Association workgroups on diagnostic guidelines for Alzheimer’s disease. Alzheimers Dement..

[3] Dubois B, Feldman HH, Jacova C, Hampel H, Molinuevo JL, Blennow K (2014). Advancing research diagnostic criteria for Alzheimer’s disease: the IWG-2 criteria. Lancet Neurol..

[4] Mattsson N, Zetterberg H, Hansson O, Andreasen N, Parnetti L, Jonsson M (2009). CSF biomarkers and incipient Alzheimer disease in patients with mild cognitive impairment. JAMA..

[5] Mulder C, Verwey NA, van der Flier WM, Bouwman FH, Kok A, van Elk EJ (2010). Amyloid- (1-42), total tau, and phosphorylated tau as cerebrospinal fluid biomarkers for the diagnosis of Alzheimer disease. Clin Chem..

[6] Duits FH, Teunissen CE, Bouwman FH, Visser P-J, Mattsson N, Zetterberg H (2014). The cerebrospinal fluid “Alzheimer profile”: easily said, but what does it mean?. Alzheimer’s Dement.

[7] Toledo JB, Zetterberg H, van Harten AC, Glodzik L, Martinez-Lage P, Bocchio-Chiavetto L (2015). Alzheimer’s disease cerebrospinal fluid biomarker in cognitively normal subjects. Brain..

[8] Mirra SS. The CERAD neuropathology protocol and consensus recommendations for the postmortem diagnosis of Alzheimer’s disease: a commentary. Neurobiol Aging. 18 4 Suppl:S91–4. http://www.ncbi.nlm.nih.gov/pubmed/9330994. Accessed 9 Sept 2019. 10.1016/s0197-4580(97)00058-4 9330994

[9] Knopman DS, DeKosky ST, Cummings JL, Chui H, Corey-Bloom J, Relkin N (2001). Practice parameter: diagnosis of dementia (an evidence-based review): report of the Quality Standards Subcommittee of the American Academy of Neurology. Neurology..

[10] Beach TG, Monsell SE, Phillips LE, Kukull W (2012). Accuracy of the clinical diagnosis of Alzheimer disease at National Institute on Aging Alzheimer Disease Centers, 2005–2010. J Neuropathol Exp Neurol..

[11] Degerman Gunnarsson M, Ingelsson M, Blennow K, Basun H, Lannfelt L, Kilander L (2016). High tau levels in cerebrospinal fluid predict nursing home placement and rapid progression in Alzheimer’s disease. Alzheimers Res Ther..

[12] De Meyer G, Shapiro F, Vanderstichele H, Vanmechelen E, Engelborghs S, De Deyn PP (2010). Diagnosis-independent Alzheimer disease biomarker signature in cognitively normal elderly people. Arch Neurol..

[13] Bertens D, Tijms BM, Scheltens P, Teunissen CE, Visser PJ (2017). Unbiased estimates of cerebrospinal fluid β-amyloid 1-42 cutoffs in a large memory clinic population. Alzheimers Res Ther..

[14] Zwan M, van Harten A, Ossenkoppele R, Bouwman F, Teunissen C, Adriaanse S (2014). Concordance between cerebrospinal fluid biomarkers and [11C] PIB PET in a memory clinic cohort. J Alzheimers Dis..

[15] Zwan MD, Rinne JO, Hasselbalch SG, Nordberg A, Lleó A, Herukka S-K (2016). Use of amyloid-PET to determine cutpoints for CSF markers. Neurology..

[16] Palmqvist S, Zetterberg H, Blennow K, Vestberg S, Andreasson U, Brooks DJ (2014). Accuracy of brain amyloid detection in clinical practice using cerebrospinal fluid β-amyloid 42. JAMA Neurol..

[17] Blennow K, Hampel H (2003). CSF markers for incipient Alzheimer’s disease. Lancet Neurol..

[18] Blennow K, Hampel H, Weiner M, Zetterberg H (2010). Cerebrospinal fluid and plasma biomarkers in Alzheimer disease. Nat Rev Neurol..

[19] van der Flier WM, Scheltens P (2018). Amsterdam dementia cohort: performing research to optimize care. J Alzheimers Dis..

[20] Jongbloed W, Kester MI, van der Flier WM, Veerhuis R, Scheltens P, Blankenstein MA (2013). Discriminatory and predictive capabilities of enzyme-linked immunosorbent assay and multiplex platforms in a longitudinal Alzheimer’s disease study. Alzheimers Dement..

[21] Shaw LM, Vanderstichele H, Knapik-Czajka M, Clark CM, Aisen PS, Petersen RC (2009). Cerebrospinal fluid biomarker signature in Alzheimer’s disease neuroimaging initiative subjects. Ann Neurol..

[22] Verhage F (1964). Intelligentie en Leeftijd: Onderzoek bij Nederlanders van Twaalf tot Zevenenzeventig Jaar [Intelligence and Age: Study with Dutch People from Age 12 to 77].

[23] Blennow K, Shaw LM, Stomrud E, Mattsson N, Toledo JB, Buck K (2019). Predicting clinical decline and conversion to Alzheimer’s disease or dementia using novel Elecsys Aβ (1–42), pTau and tTau CSF immunoassays. Sci Rep..

[24] Iqbal K, Liu F, Gong C-X (2016). Tau and neurodegenerative disease: the story so far. Nat Rev Neurol..

[25] Chhatwal JP, Schultz AP, Marshall GA, Boot B, Gomez-Isla T, Dumurgier J (2016). Temporal T807 binding correlates with CSF tau and phospho-tau in normal elderly. Neurology..

[26] Mattsson N, Schöll M, Strandberg O, Smith R, Palmqvist S, Insel PS (2017). 18 F-AV-1451 and CSF T-tau and P-tau as biomarkers in Alzheimer’s disease. EMBO Mol Med..

[27] Gordon BA, Friedrichsen K, Brier M, Blazey T, Su Y, Christensen J (2016). The relationship between cerebrospinal fluid markers of Alzheimer pathology and positron emission tomography tau imaging. Brain..

[28] Pooler AM, Phillips EC, Lau DHW, Noble W, Hanger DP (2013). EMBO Reports.

[29] Sato C, Barthélemy NR, Mawuenyega KG, Patterson BW, Gordon BA, Jockel-Balsarotti J (2018). Tau kinetics in neurons and the human central nervous system. Neuron.

[30] Sutphen CL, Jasielec MS, Shah AR, Macy EM, Xiong C, Vlassenko AG (2015). Longitudinal cerebrospinal fluid biomarker changes in preclinical Alzheimer disease during middle age. JAMA Neurol..

[31] Lleó A, Alcolea D, Martínez-Lage P, Scheltens P, Parnetti L, Poirier J, et al. Longitudinal cerebrospinal fluid biomarker trajectories along the Alzheimer’s disease continuum in the BIOMARKAPD study. Alzheimers Dement. 2019; 10.1016/j.jalz.2019.01.015. 10.1016/j.jalz.2019.01.015 30967340

[32] Wildsmith KR, Schauer SP, Smith AM, Arnott D, Zhu Y, Haznedar J (2014). Identification of longitudinally dynamic biomarkers in Alzheimer’s disease cerebrospinal fluid by targeted proteomics. Mol Neurodegener..

[33] van Rossum IA, Vos SJB, Burns L, Knol DL, Scheltens P, Soininen H (2012). Injury markers predict time to dementia in subjects with MCI and amyloid pathology. Neurology..

[34] Kester MI, van der Vlies AE, Blankenstein MA, Pijnenburg YAL, van Elk EJ, Scheltens P (2009). CSF biomarkers predict rate of cognitive decline in Alzheimer disease. Neurology..

[35] Jansen WJ, Ossenkoppele R, Knol DL, Tijms BM, Scheltens P, Verhey FRJ (2015). Prevalence of cerebral amyloid pathology in persons without dementia. JAMA..

[36] Slot RER, Kester MI, Van Harten AC, Jongbloed W, Bouwman FH, Teunissen CE (2019). ApoE and clusterin CSF levels influence associations between APOE genotype and changes in CSF tau, but not CSF Aβ42, levels in non-demented elderly. Neurobiol Aging..

[37] Konijnenberg E, Tijms BM, Gobom J, Dobricic V, Bos I, Vos S (2020). APOE ϵ4 genotype-dependent cerebrospinal fluid proteomic signatures in Alzheimer’s disease. Alzheimers Res Ther..

[38] Reus LM, Stringer S, Posthuma D, Teunissen CE, Scheltens P, Pijnenburg YAL (2020). Degree of genetic liability for Alzheimer’s disease associated with specific proteomic profiles in cerebrospinal fluid. Neurobiol Aging.

