# Supplementary material for: Four subgroups based on tau levels in Alzheimer’s disease observed in two independent cohorts
Source: Alzheimers Res Ther. 2021 Jan 4;13:2. doi: 10.1186/s13195-020-00713-3 (PMC7780683; doi:10.1186/s13195-020-00713-3)
Supplement: Supplementary file 1 — Additional file 1. [file 13195_2020_713_MOESM1_ESM.docx]

**Supplementary methods**

*2.1. Amsterdam dementia cohort*

*2.1.1. Subjects*

We selected all patients with subjective cognitive decline (considered as NC), mild cognitive impairment (MCI) or AD dementia, who had baseline CSF tau measurements available and visited our memory clinic between November 2000 and December 2016. This resulted in 2724 patients. Subjects underwent extensive cognitive screening at baseline, with physical and neurological examination, EEG, MRI, and laboratory tests. Neuropsychological investigation included at least one test per cognitive domain, as well as Mini Mental State Examination (MMSE) for global cognition. The Verhage scale was used for assessment of educational level.^22^ Diagnoses were made by consensus in a multidisciplinary team without knowledge of CSF results. AD dementia and MCI were diagnosed according to the criteria of the National Institute of Neurological and Communicative Disorders and Stroke-Alzheimer’s Disease and Related Disorders association (NINCDS-ADRDA) and Petersen’s criteria respectively,^35,36^ and all patients met the core clinical NIA-AA criteria.^2,37^ When all clinical investigations were normal (i.e. criteria for MCI or any psychiatric of neurological disorder not fulfilled), patients were labeled as NC. At follow-up visits physical, neurological and neuropsychological examinations were repeated. All subjects gave written informed consent for the use of their clinical data and CSF for research purposes. The study was approved by the ethical review board of the VU University Medical Center.

*2.1.2. CSF analysis of AD biomarkers*

CSF was obtained by lumbar puncture using a 25-gauge needle and a syringe, and collected in 10 mL polypropylene tubes (Sarstedt, Nümbrecht, Germany). Within two hours, the remaining CSF was centrifuged at 1800*g* for 10 minutes at 4°C, transferred to new polypropylene tubes and stored either at -20°C until analysis of Aβ42, tau and p-tau, or directly at -80°C until further analysis. The team involved in CSF analyses was blinded for clinical diagnosis. Aβ42 and total tau were measured with commercially available ELISAs (β-amyloid_(1-42)_, and hTAU-Ag; Fujirebio, Ghent, Belgium) on a routine basis as described before.^20^ Measurements took place consecutively within one month of the patient’s baseline visit. Intra-assay coefficients of variation (CV) were (mean±SD) 2.0±0.5% for Aβ42, 3.2±1.3% for tau and 2.9±0.8% for p-tau, inter-assay CVs (mean±SD) were 10.9±1.8% for Aβ42, and 9.9±2.1% for tau. As it is known that Aβ42 levels have increased over time as measured with the ELISA used in our cohort,^38^ we used rescaled values of Aβ42 as developed recently by our group.^38^

*2.1.3. APOE genotyping*

For Apolipoprotein E (APOE) genotyping, DNA was isolated from 10 mL EDTA blood by the QIAamp DNA blood isolation kit from Qiagen. The genotype was determined with the Light Cycler APOE mutation detection kit (Roche Diagnostics GmbH, Mannheim, Germany).

*2.2. Replication cohort*

Data were obtained from the Alzheimer’s Disease Neuroimaging Initiative (ADNI) database (adni.loni.usc.edu). The primary goal of ADNI is to test whether serial magnetic resonance imaging (MRI), positron emission tomography (PET), other biological markers, and clinical and neuropsychological assessment can be combined to measure the progression of mild cognitive impairment (MCI) and early Alzheimer’s disease (AD). The Principal Investigator of this initiative is Michael W. Weiner, MD, VA Medical Center and University of California–San Francisco. For up-to-date information, see www.adni-info.org.

*2.2.1. Subjects*

We selected 1221 individuals from ADNI phase-1, phase-2 and GO with normal cognition (n=371), MCI (n=622) or dementia (n=228) when they had baseline CSF biomarkers available. We used clinical data of baseline or screening visits and follow-up visits (diagnosis, MMSE scores, repeated CSF measures) if available.

2.2.2. CSF analysis

We used baseline and follow-up CSF data analyzed using a multiplex xMAP Luminex platform (Luminex Corp) with immunoassay kit-based reagents (INNO-BIA Alzbio3; Innogenetics) as described elsewhere.[32] Follow-up was performed annually up to 4 years. We repeated analyses for CSF t-tau and p-tau measured with the novel Elecsys platform to determine dependence of subgroups on platform. For longitudinal analyses, measures within individuals were obtained from the same batch.^39^ A subset of ADNI wave 3 subjects had both Elecsys CSF markers and tau PET available. These individuals were labelled according to cutoffs obtained from ADNI 1, 2, and GO.

2.2.3. Tau PET

Standardized uptake value Ratios (SUVr) of the different Braak regions were downloaded from the ADNI-LONI website (Details on acquisition procedures for these [^18^F]flortaucipir and MRI images can be found elsewhere (*http://adni.loni.usc.edu/methods/documents/*). [^18^F]Flortaucipir images were processed as described previously.^40^ Briefly, SUVr images were created over a time-window of 80-100 minutes post-injection, using inferior cerebellar gray as reference region. All MPRAGE T1 images were parcelated with FreeSurfer ( [*http://surfer.nmr.mgh.harvard.edu/*](http://surfer.nmr.mgh.harvard.edu/)) and [^18^F]flortaucipir standardized uptake value ratios (SUVR) images were coregistered to native T1s using SPM to derive regional SUVr for each participant. Regional SUVr values were extracted from 3 a priori defined composite regions of interest (ROIs): Braak stage I/II, III/IV and V/IV recapitulating the neuropathological Braak&Braak stages.^41,42^ Regions that were used for the Braak stages I-IV from FreeSurfer can be found in Baker et al^43^, table 2. [^18^F]-flortaucipir images were not partial volume corrected.
